# Brassinin Induces Apoptosis, Autophagy, and Paraptosis via MAPK Signaling Pathway Activation in Chronic Myelogenous Leukemia Cells

**DOI:** 10.3390/biology12020307

**Published:** 2023-02-14

**Authors:** Min Hee Yang, In Jin Ha, Seok-Geun Lee, Junhee Lee, Jae-Young Um, Gautam Sethi, Kwang Seok Ahn

**Affiliations:** 1 KHU-KIST Department of Converging Science and Technology, Kyung Hee University, Seoul 02447, Republic of Korea; 2 Korean Medicine Clinical Trial Center (K-CTC), Korean Medicine Hospital, Kyung Hee University, Seoul 02447, Republic of Korea; 3 Department of Science in Korean Medicine, Kyung Hee University, 24 Kyungheedae-ro, Dongdaemun-gu, Seoul 02447, Republic of Korea; 4 Department of Pharmacology, Yong Loo Lin School of Medicine, National University of Singapore, Singapore 117600, Singapore

**Keywords:** brassinin, apoptosis, autophagy, paraptosis, mitogen-activated protein kinase (MAPK), chronic myelogenous leukemia (CML)

## Abstract

**Simple Summary:**

Cell death was once thought of as two distinct processes involving mainly apoptosis or necrosis. In recent years, several different forms of cell death have gained importance, such as autophagy, paraptosis, and ferroptosis. Brassinin (BSN) is a phytoalexin identified from Cruciferase vegetables (Brassica) and various pharmacological properties have been reported. However, the efficacy of BSN for chronic myelogenous leukemia has not been elucidated. In our study, we confirmed whether BSN modulates the different cell death processes including apoptosis, autophagy, and paraptosis in chronic myelogenous leukemia cells. In addition, we confirmed that BSN promotes the activation of the MAPK signaling pathway. In this study, BSN could stimulate the activation of apoptosis, autophagy, and paraptosis by modulating the MAPK signaling cascade.

**Abstract:**

Brassinin (BSN), a potent phytoalexin found in cruciferous vegetables, has been found to exhibit diverse anti-neoplastic effects on different cancers. However, the impact of BSN on chronic myelogenous leukemia (CML) cells and the possible mode of its actions have not been described earlier. We investigated the anti-cytotoxic effects of BSN on the KBM5, KCL22, K562, and LAMA84 CML cells and its underlying mechanisms of action in inducing programmed cell death. We noted that BSN could induce apoptosis, autophagy, and paraptosis in CML cells. BSN induced PARP cleavage, subG1 peak increase, and early apoptosis. The potential action of BSN on autophagy activation was confirmed by an LC3 expression and acridine orange assay. In addition, BSN induced paraptosis through increasing the reactive oxygen species (ROS) production, mitochondria damage, and endoplasmic reticulum (ER) stress. Moreover, BSN promoted the activation of the MAPK signaling pathway, and pharmacological inhibitors of this signaling pathway could alleviate all three forms of cell death induced by BSN. Our data indicated that BSN could initiate the activation of apoptosis, autophagy, and paraptosis through modulating the MAPK signaling pathway.

## 1. Introduction

Chronic myelogenous leukemia (CML) is a major hematopoietic disorder marked by the presence of the Philadelphia chromosome (Ph), which is generated when chromosomes 9 and 22 break and exchange their parts [1,2]. This alteration can result in the aberrant activation of Bcr-abl kinase [3,4], which in turn can regulate the aberrant tumorigenesis through stimulating the initiation of diverse signal transduction pathways, including a mitogen-activated protein kinase (MAPK), and a Janus kinase/signal transducer and activator of transcription (JAK/STAT), and the phosphatidylinositol 3 kinase (PI3K) signaling pathway, and the nuclear factor-κB (NF-κB) [5,6,7,8,9]. Imatinib mesylate is the primary tyrosine kinase that has been used to target Bcr-abl and has been clinically approved for the management of CML [10,11]. Unfortunately, disease relapse has been reported frequently as a result of resistance to imatinib therapy [12,13,14]. A number of studies have indicated the necessity to develop other novel targets as well pharmacological strategies to control this lethal disease [12,15,16,17].

Cell death acts as a crucial role in the pathogenesis of several diseases [18]. Apoptosis or necrosis were considered, which occupied the greatest area of cell death [18,19]. Apoptosis is a programmed process leading to cellular degradation, which is primarily characterized by the enzymatic cleavage of poly (ADP-ribose) polymerase (PARP) by caspase-3 [20,21]. In recent years, several other cell death forms gained importance, including paraptosis, autophagy, pyroptosis, and ferroptosis [22,23,24,25]. Interestingly, recent studies have demonstrated that diverse cell death processes can be initiated as a result of specific alterations in the tumor cells, such as the formation of apoptotic bodies in apoptosis, autophagosomes during autophagy, and large-scale vacuoles in the cytoplasm of undergoing paraptosis [26,27]. Autophagy predominantly causes the rapid degradation of the damaged cytoplasmic proteins, which are enclosed in autophagosomes and thereafter degraded in autophagolysosomes [28]. The different representative characteristics of paraptosis are endoplasmic reticulum (ER) and mitochondrial swelling [29,30,31], and a number of larger vacuoles are created due to their fusion [30,32,33]. Recent reports have identified that Alix can serve as a novel inhibitor of paraptosis [34,35]. A number of natural compounds can exert their anti-cancer effects through inducing these cell death processes [16,36,37,38].

A number of natural agents play an important role in eliminating tumor cells through different cell death mechanisms [39,40,41]. Brassinin (BSN), a phytoalexin isolated from Chinese cabbage, has exhibited potent anti-proliferative and anti-carcinogenic actions against different malignancies [42,43,44,45]. For instance, our group has previously reported that BSN can stimulate apoptosis by regulating JAK/STAT3 and PI3K/Akt/mTOR simultaneously in colorectal cancer [43]. Additionally, BSN can influence the epithelial-mesenchymal transition (EMT) process through downregulating the PI3K/Akt/mTOR signaling cascade in lung carcinoma cells [42]. Hong et al. established that BSN can modulate proliferation and apoptosis via the regulation of the PI3K/MAPK signaling pathway and stimulate ROS generation [45]. Despite being a potential anti-cancer agent, the effects and molecular mechanisms of BSN on the CML model have not yet been elucidated surprisingly. In this study, we have examined whether BSN can impart anti-neoplastic effects against a wide variety of CML cells. We focused on the influence of BSN in modulating the different cell death processes, including apoptosis, autophagy, and paraptosis. We also noted that the BSN-induced MAPK signaling pathway activation could effectively mediate the apoptosis, autophagy, and paraptosis activation in CML cells.

## 2. Materials and Methods

### 2.1. Reagents

Brassinin (BSN) was purchased from LKT laboratories (Minneapolis, MN, USA). Antibodies of Bax, PARP, Bcl-2, IAP-1, ATF4, CHOP, and β-actin were purchased from Santa Cruz Biotechnology (Santa Cruz, CA, USA). Antibodies of Alix, LC3, Atg7, p-Beclin-1, Beclin-1, p-JNK, JNK, p-p38, p-ERK, and ERK were purchased from Cell Signaling Technology (Beverly, MA, USA).

### 2.2. Cell Culture

Human chronic myeloid leukemia (CML) K562, KBM5, LAMA84, and KCL22 cells were purchased from American Type Culture Collection (Manassas, VA, USA). KBM5 cells were cultured in IMDM medium. Other cells were cultured in RPMI1640 medium. The medium contained 10% fetal bovine serum (FBS) and 1% penicillin-streptomycin, and the cells were maintained at 37 °C under 5% CO_2_ atmosphere.

### 2.3. MTT Assay

KBM5, K562, KCL22, LAMA84 cells and human peripheral blood mononuclear cells (PBMCs) were treated with BSN (0, 10, 15, 30, 50, 100 μM) for 24 h and cell viability was measured by MTT assay as described before [36]. KBM5, K562, KCL22, and LAMA84 cells were treated with BSN (0, 10, 15, 30, 50, 100 μM) for 24 h. The absorbance was measured by VARIOSKAN LUX (Thermo Fisher Scientific Inc, Waltham, MA, USA) at 570 nm. The cell viability was normalized as relative percentage in comparison with non-treated controls. The half-inhibitory concentration (IC_50_) calculation was performed after MTT assay, using the linear (y = mx + n) equation.

### 2.4. Western Blot Analysis

The various CML cells were incubated at indicated concentrations and time, and Western blot analysis for detection of specific antibodies was performed as elaborated earlier [37]. Whole cell lysates were prepared, and equal amount of proteins was resolved in SDS-PAGE gel and transferred into the nitrocellulose membrane. Then, membranes were blocked with 5% skimmed milk for 1 h and probed with specific primary antibodies (1:3000) overnight. Next day, secondary antibodies (1:5000) were added for 1 h and membranes were detected with enhanced chemiluminescence (ECL, EZ-Western Lumi Femto, DOGEN).

### 2.5. Live and Dead Assay

CML cells were treated with BSN 50 μM for 24 h and apoptosis was examined by live and dead assay as described previously [36]. The cells were stained with 1 μL/mL of Calcein AM and 2 μL/mL of Ethd-1 at 37 °C for 30 min (Invitrogen, Carlsbad, CA, USA). The cells were attached on slide glass by cytospin. The fluorescence signals were detected by Olympus FluoView FV1000 confocal micro-scope (Tokyo, Japan).

### 2.6. Immunocytochemistry

CML cells were incubated with BSN 50 μM for 24 h and expression of LC3 was analyzed by immunocytochemistry as indicated earlier [36]. The cells were fixed with 4% paraformaldehyde (PFA) at the room temperature and incubated with 0.2% Triton-X100. They were blocked with 5% BSA for 1 h and incubated with anti-LC3 (1:100) overnight at 37 °C. Next day, cells were incubated with Alexa Fluor^®^ 594 donkey anti-rabbit IgG (H + L) antibody (1:1000) for 1 h and incubated with DAPI for 3 min. The fluorescence signal was detected by Olympus FluoView FV1000 confocal micro-scope (Tokyo, Japan).

### 2.7. Cell Cycle Analysis

The cells were treated with BSN 50 μM for 24 h and cell cycle analysis was performed to determine the effect of BSN on cell cycle progression as reported previously [37]. After treatment, the cells were harvested and fixed with EtOH overnight. Next day, cells resuspended with RNase A for 1 h at 37 °C and stained with propidium iodide (PI). The cells were analyzed by BD AccuriTM C6 Plus Flow Cytometer (BD Biosciences, Becton-Dickinson) with BD Accuri C6 Plus software.

### 2.8. Annexin V Assay

To determine apoptosis, Annexin V assay was performed as described before [43]. KBM5, K562, KCL22, and LAMA84 cells were treated with 50 μM of BSN for 24 h. Then, cells were harvested and stained with FITC-tagged Annexin B antibody and PI for 15 min at room temperature. The cells were analyzed by BD Accuri^TM^ C6 Plus Flow Cytometer (BD Biosciences, Becton-Dickinson, Franklin Lakes, NJ, USA) with BD Accuri C6 Plus software.

### 2.9. Acridine Orange Assay

CML cells were treated with BSN 50 μM for 24 h and acridine orange assay was performed as described earlier [16]. The cells were stained with acridine orange for 20 min at 37 °C and detected by BD Accuri^TM^ C6 Plus Flow Cytometer (BD Biosciences, Becton-Dickinson, Franklin Lakes, NJ, USA) with BD Accuri C6 Plus software.

### 2.10. Mesurement of Reactive Oxygen Species (ROS)

The cells were treated with NAC (1 mM), H_2_O_2_ (200 μM), or BSN and incubated with 2′,7′-dichlorofluorescin diacetate (H_2_DCF-DA). Then, the stained cells were detected by BD Accuri^TM^ C6 Plus Flow Cytometer (BD Biosciences, Becton-Dickinson, Franklin Lakes, NJ, USA) with BD Accuri C6 Plus software [43].

### 2.11. Mitochonrial Membrane Potential Assay

The cells were treated with BSN 50 μM and stained with TMRE 50 nM for 30 min at 37 °C. Thereafter, the cells were detected by BD Accuri^TM^ C6 Plus Flow Cytometer (BD Biosciences, Becton-Dickinson, Franklin Lakes, NJ, USA) with BD Accuri C6 Plus software [46].

### 2.12. ER Stress Assay

KBM5, K562, KCL22, and LAMA84 cells were incubated with BSN and cells were stained by ER-Tracker Red (1 μM) for 30 min and DAPI (10 μg/mL) for 30 min. Then, the cells were detected by Olympus FluoView FV1000 confocal micro-scope (Tokyo, Japan).

### 2.13. GSH/GSSG Assay

The cellular GSH/GSSG ratio was evaluated by GSH/GSSG-Glo^TM^ Assay kit (Promega, Madison, WI, USA) according to the manufacturer’s protocol and as described before [43].

### 2.14. Isolation of Human Peripheral Blood Mononuclear Cells (PBMCs)

Human peripheral blood mononuclear cells (PBMCs) were isolated from the blood healthy adult donors (volunteers) by density gradient centrifugation on a lymphoprep (Axis-Shield PoCAS, Oslo, Norway).

### 2.15. Statistical Analysis

All the numerical values have been represented as the mean ± SD. The statistical significance of the data compared with the untreated control was determined using the Student’s unpaired *t*-test. Significance was set at * *p* < 0.05, ** *p* < 0.01, and *** *p* < 0.001. All the experiments were repeated at least thrice to ensure the reproducibility.

## 3. Results

### 3.1. BSN Substantially Suppressed the Cell Viability and Induced Cell Death

**Figure 1:**
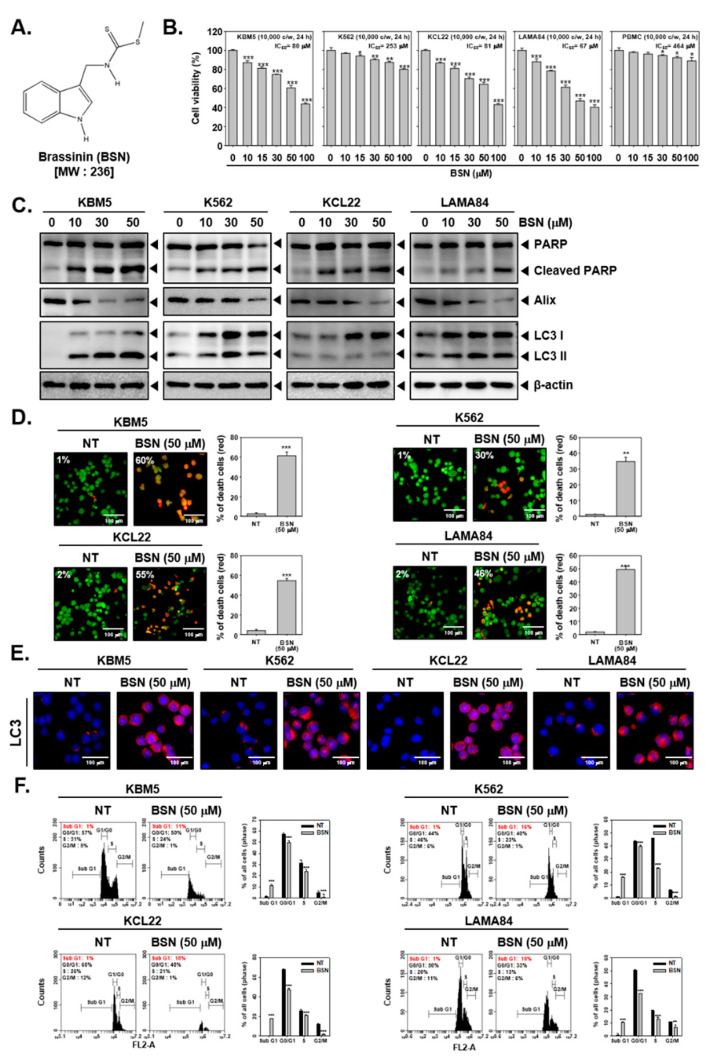
BSN induces cell death. (**A**) The chemical structure of Brassinin (BSN). (**B**) KBM5, K562, KCL22, and LAMA84 cells were treated with BSN (0, 10, 15, 30, 50, 100 μM) for 24 h and MTT assay was performed. * *p* < 0.05 vs. non-treated (NT) cells, ** *p* < 0.01 vs. non-treated (NT) cells, and *** *p* < 0.001 vs. non-treated (NT) cells. (**C**) The cells were treated with BSN as described above panel B, and immunoblotting was conducted. (**D**) The cells were treated with BSN (50 μM) for 24 h and live and dead assay was conducted. (**E**) The cells were treated as described above in panel D; expression of LC3 was elaborated with immunocytochemistry. (**F**) The cells were treated as described above in panel D; apoptotic cells were analyzed via cell cycle analysis. Data represent mean ± SD. *** *p* < 0.001 vs. non-treated (NT) cells. The results shown are representative of the three independent experiments.

The structure of BSN is depicted in Figure 1A. The cytotoxic action of BSN against CML cells and (PBMCs) were examined by an MTT assay. We noted that BSN significantly suppressed the viability in the KBM5, KCL22, and LAMA84 cells, but it was only slightly affected in the K562 cells compared to other cells (Figure 1B). In addition, the results showed that BSN was more cytotoxic toward CML cells rather than PBMCs. We found that BSN exhibited less than 10% cytotoxicity against PBMCs at a concentration up to 50 μM. Next, we examined the various modes of the cell death pathways activated upon exposure to BSN. The CML cells were treated with BSN (0, 10, 15, 30, and 50 μM) for 24 h and a Western blot analysis for apoptosis-, autophagy-, and paraptosis-related markers was conducted. As shown in Figure 1C and Figures S1, S12 and S13, both apoptosis and autophagy were stimulated through promoting the PARP cleavage as well as the LC3 expression in all the CML cells exposed to BSN at a 50 μM concentration. Surprisingly, BSN caused paraptosis through downregulating the expression of Alix, a well-characterized marker of paraptosis that attenuates the onset of this process. Therefore, we selected 50 μM, which could target all CML cells but displayed a low cytotoxicity against PBMCs, as the highest concentration. Next, we examined the action of BSN on cell death using a live and dead assay. As shown in Figure 1D, the BSN treatment attenuated the viability of the CML cells. Moreover, as shown in Figure 1E, the BSN-treated cells exhibited a higher expression on LC3. Additionally, BSN enhanced the distribution of the cells in the sub G1 phase (Figure 1F).

### 3.2. BSN Promoted Apoptotic Cell Death

**Figure 2:**
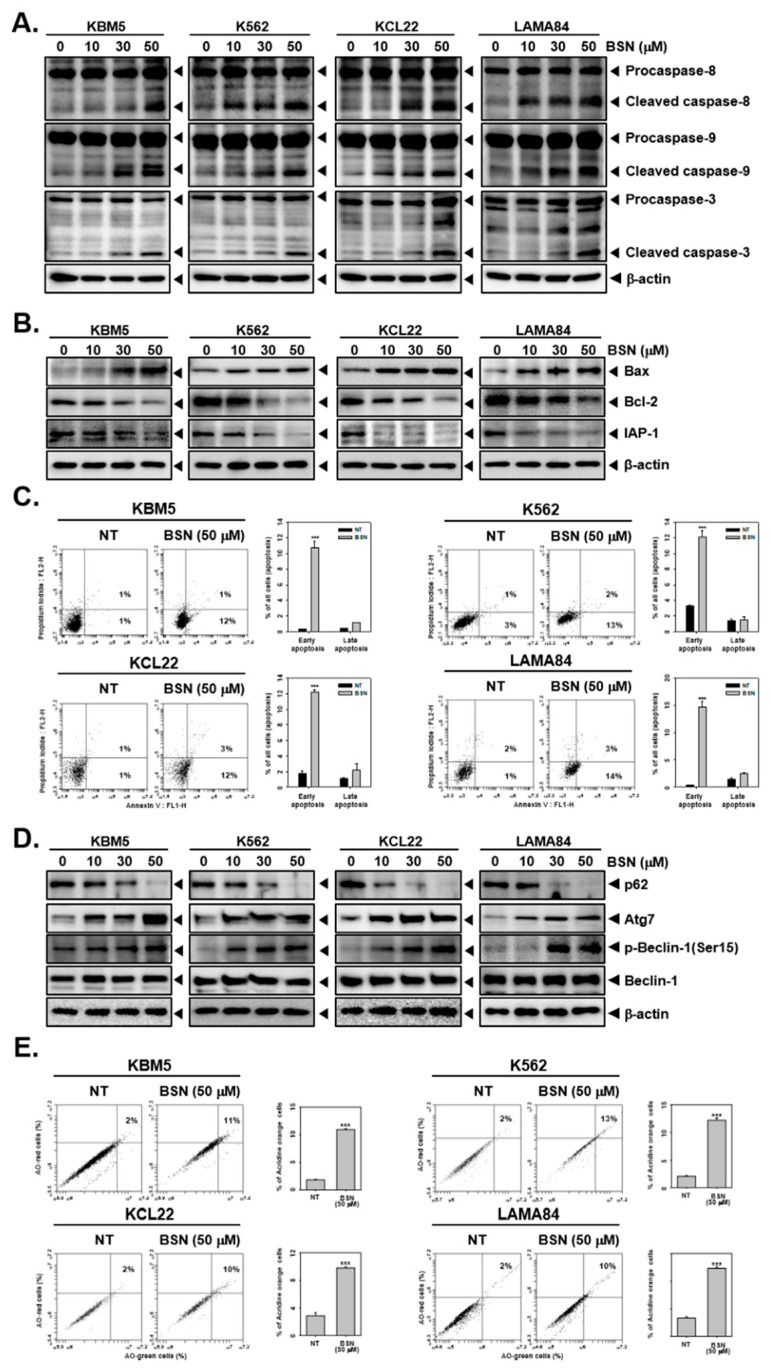
BSN induces both apoptosis and autophagy. (**A**,**B**) KBM5, K562, KCL22, and LAMA84 cells were incubated with BSN (0, 10, 15, 30, 50, 100 μM) for 24 h and immunoblotting was carried out. (**C**) The cells were treated with BSN (50 μM) for 24 h and apoptotic cells were analyzed by Annexin V assay. (**D**) The cells were treated as described above in panel A; Western blotting for various proteins was carried out. (**E**) The cells were treated as described above in panel B; autophagy was measured by acridine orange assay. Data represent mean ± SD. *** *p* < 0.001 vs. non-treated (NT) cells. The results shown are representative of the three independent experiments.

Next, the impact of BSN on apoptotic cell death was determined through Western blotting and Annexin V assays. Our data showed that BSN promoted the caspase-8, caspase-9, and caspase-3 activation (Figure 2A and Figures S2, S14 and S15) and thus promoted apoptosis. As shown in Figure 2B and Figures S3, S16 and S17, BSN augmented the expression of Bax but reduced that of the Bcl-2 and IAP-1 proteins. In addition, the cells were classified into distinct stages such as necrosis, early apoptosis, late apoptosis, and live cells through employing the Annexin V assay. Surprisingly, the BSN-treated cells showed an increased concentration in the early apoptosis stage (Figure 2C). The results suggested that BSN can lead to pronounced cell death.

### 3.3. BSN Induced Substantial Autophagy

We next examined the action of BSN on the autophagy activation in CML cells by a Western blot analysis and acridine orange assays. As shown in Figure 2D and Figures S4, S18 and S19, we noticed that the cells exposed to BSN showed a decreased p62 expression but increased that of the Atg7, p-Beclin-1, and Beclin-1 proteins. In addition, as depicted in Figure 2E, the percentage of the autophagosome-stained cells markedly increased, and based on these results, it could be concluded that autophagy could be effectively stimulated upon BSN exposure.

### 3.4. BSN Induced Paraptosis and ER Stress

**Figure 3:**
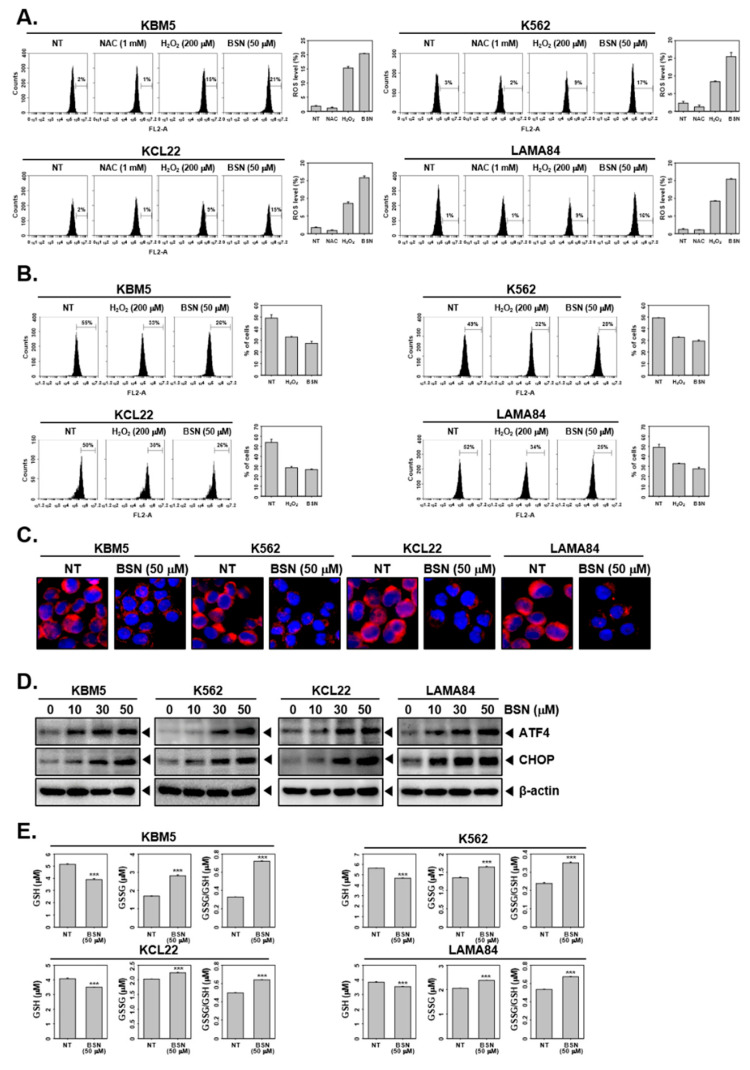
BSN induces ER stress in CML cells. (**A**) KBM5, K562, KCL22, and LAMA84 cells were treated with NAC (1 mM), H_2_O_2_ (200 μM), or BSN (50 μM) for 12 h and ROS production was measured. (**B**) The cells were treated with H_2_O_2_ (200 μM) or BSN (50 μM) for 24 h and mitochondrial membrane potential was analyzed by flow cytometer. (**C**) Cells were treated as described in panel B; ER-Tracker Red and DAPI staining to analyze ER structure and cell nuclear. (**D**) The cells were treated with BSN for indicated concentrations for 24 h and immunoblotting for ATF4, CHOP, and β-actin was carried out. (**E**) The cells were treated described in panel B, and GSH/GSSG assay was performed. Data represent mean ± SD. *** *p* < 0.001 vs. non-treated (NT) cells. The results shown are representative of the three independent experiments.

We examined whether BSN induced changes in the ROS production and mitochondrial membrane potential. First, the ROS levels were measured through H_2_DCF-DA staining. As shown in Figure 3A, the antioxidant NAC prevents ROS production and H_2_O_2_ increased the ROS production. The BSN treatment significantly increased the ROS levels. In addition, the loss of mitochondrial membrane potential was observed in H_2_O_2_- or BSN-treated cells (Figure 3B). Subsequently, the CML cells were treated with BSN and stained by ER-Tracker Red to monitor the expression of the key transcription factors involved in the ER stress pathway. As shown in Figure 3C, the staining of the ER indicated that vacuolization was obtained primarily from the ER. BSN induced a marked expression of ATF4 and CHOP, two major transcription factors of the ER stress pathway (Figure 3D and Figures S5 and S20). Additionally, we explored the impact of BSN on the GSH/GSSG system to analyze the mechanisms driving the ROS production. As shown in Figure 3E, the depletion of GSH was observed contrariwise, and the GSSG and GSSG/GSH ratio was increased, indicating the existence of oxidative stress.

### 3.5. BSN Induced Cell Death through Affecting MAPK Activation

**Figure 4:**
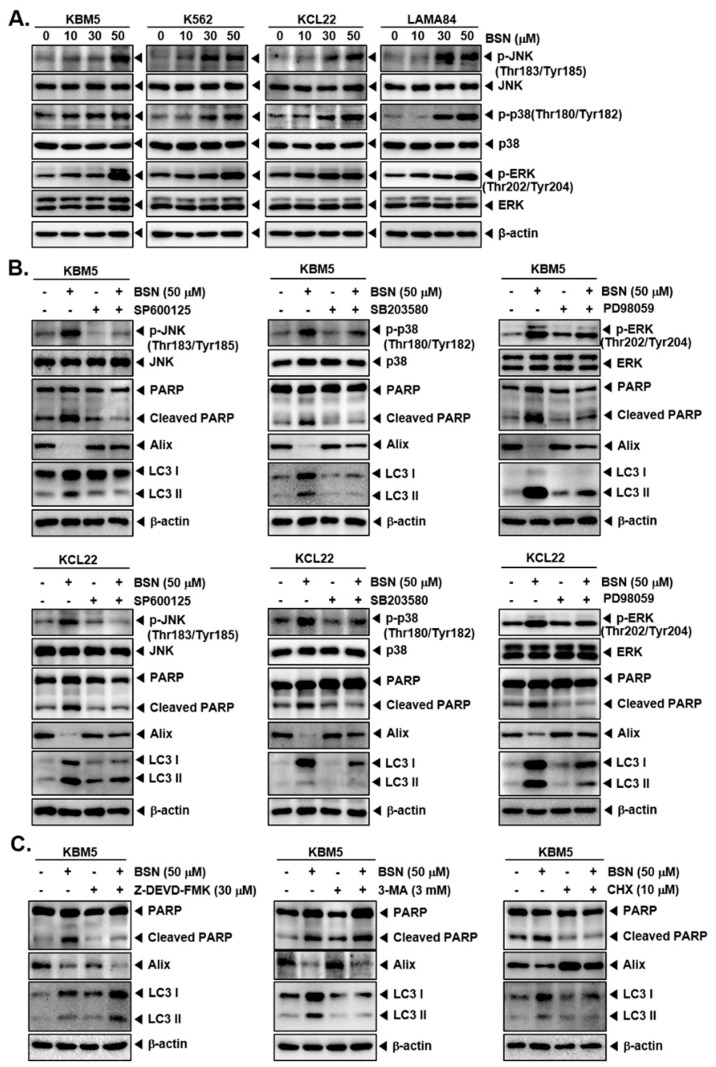
BSN activates MAPK signaling pathway. (**A**) KBM5, K562, KCL22, and LAMA84 cells were treated with BSN (0, 10, 15, 30, 50, 100 μM) for 24 h and Western blotting was carried out. (**B**) The cells were treated with SP600125 (10 μM) or SB203580 (10 μM) or PD98059 (10 μM) and treated with BSN (50 μM) for 24 h. Western blot analysis was performed. (**C**) The cells were treated with Z-DEVD-FMMK (30 μM) or 3-MA (3 mM) or CHX (10 μM) and treated with BSN (50 μM) for 24 h. Then, expressions of various proteins were evaluated by Western blot analysis. The results shown are representative of the three independent experiments.

The MAPK signaling pathway plays an important role in the regulation of various hallmarks of cancer; hence, we examined the action of BSN on this pathway. As shown in Figure 4A (Figures S6 and S21–S23), the phosphorylation of JNK, p38, and ERK was significantly increased by BSN. Then, we examined whether MAPKs suppression can affect BSN-induced apoptosis, autophagy, and paraptosis by MAPK inhibitors. As shown in Figure 4B and Figures S7–S10 and S24–S29, a blockage of JNK, p38, and ERK using pharmacological blockers could reverse the BSN-mediated upregulation of the PARP cleavage and LC3, as well as the BSN-stimulated downregulation of Alix. These results suggested a critical role of the MAPK signaling pathway in the regulation of the cell death caused by BSN.

### 3.6. Inhibitors of Apoptosis, Autophagy, and Paraptosis Attenuated BSN-Related Cell Death

We also employed Z-DEVD-FMK, a caspase-3 blocker; 3-MA, an autophagy inhibitor; and cycloheximide (CHX), a paraptosis inhibitor, to elucidate the specificity of the BSN-initiated cell death mechanisms. As shown in Figure 4C and Figures S11, S30 and S31, co-treatment of Z-DEVD-FMK and BSN inhibited the PARP cleavage, whereas co-treatment of Z-DEVD-FMK and BSN enhanced the BSN-upregulated LC3 expression and suppressed the BSN-downregulated Alix expression. The co-treatment of 3-MA and BSN inhibited the LC3 activation, increased the BSN-induced PARP cleavage, and suppressed the BSN-downregulated Alix expression. Moreover, the co-treatment of CHX and BSN attenuated the BSN-suppressed Alix expression and PARP cleavage, and the LC3 activation.

## 4. Discussion

**Figure 5:**
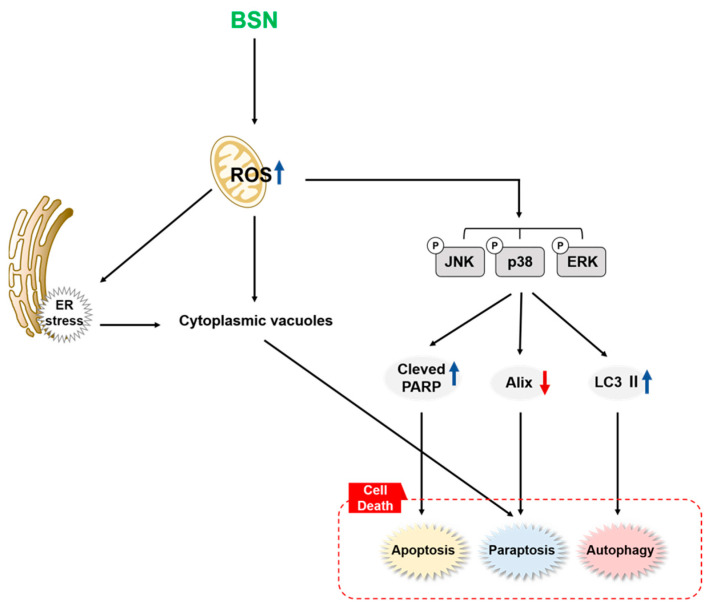
A schematic diagram showing mode of action of BSN.

BSN is a precursor of phytoalexins that can display diverse pharmacological properties [42,44,45,47,48,49]. For example, BSN was reported to cause apoptosis by modulating the PI3K/Akt/mTOR cascade in prostate cancer cells [47]. Moreover, BSN promoted apoptosis through regulating JAKs/STAT3 and PI3K/Akt/mTOR phosphorylation [48]. Surprisingly, no prior studies have compared its impact on various forms of cell death and its possible anti-cancer effects in CML. In this study, we have focused on the effect of BSN on three major forms of cell death in KBM5, KCL22, K562, and LAMA84 CML cells. We also demonstrated that BSN-induced MAPK signaling pathway activation could mediate apoptosis, autophagy, and paraptosis effectively (Figure 5).

Apoptosis plays a key role in eliminating damaged cells and thereby preventing their oncogenic transformation [50,51,52]. Apoptosis has been closely found to be involved in cancer therapy as it is a vital target of many treatment strategies [52,53,54]. Previous studies have reported that BSN exerts anti-neoplastic effects through stimulating apoptosis in solid tumor models [45,47,48,55]. We first confirmed the cytotoxicity of BSN against KBM5, KCL22, K562, and LAMA84 CML cells. BSN generally exhibited a cytotoxic effect toward CML cells. Based on the cytotoxic results, the highest concentration of 50 μM BSN was selected for further experiments. We evaluated the influence of BSN on apoptosis through using different biochemical assays. We found that BSN increased the apoptotic cell death, and the sub G1 peak increase was validated through a live and dead assay and cell cycle analysis. The Annexin V assay indicated that the BSN treatment stimulated early apoptosis in CML cells. Because the cleavage of PARP is one of the major events observed during apoptosis [56,57], we confirmed that BSN induced the expression of PARP cleavage. Moreover, BSN increased the expression of Bax but mitigated that of Bcl-2 and IAP-1. These results suggested that BSN can induce apoptosis in CML cells.

Autophagy is a conserved process involved in maintaining the optimal functioning of cells by driving the elimination of mutated or disrupted cells [28,58]. Whether autophagy is tumor suppressive or oncogenic is still controversial, and several reports have described different agents that cause autophagy in CML cells [16,58,59]. Liu et al. reported that pristimerin inhibited the cell proliferation through triggering autophagy and apoptosis in CML cells [59]. Pyrimethamine induced an interaction between the apoptosis and autophagy activation in CML cells [16]. Autophagic cells were analyzed by different biochemical assays. In addition, the levels of autophagosome-related proteins such as Atg7 and Beclin-1 were examined [60,61]. Through these results, we established that BSN induced autophagy as well as apoptosis.

Paraptosis is a newly discovered cell death process and is identified by cytoplasmic vacuoles derived from the ER [27,62]. Alix, a specific marker of paraptosis, is a negative regulator of paraptosis [34]. Our result indicated that BSN suppressed the expression of Alix. The level of ROS can play a dual role in regulating tumorigenesis [63]. For instance, a previous study has elegantly reported that homobrassinin (N-[2-(indol-3-yl)ethyl]-S-methyldithiocarbamate) can induce cell death via stimulating the ROS production and mitochondrial dysfunction [49]. We noted that BSN promoted the ROS production which was similar to other related reports [64]. The abnormal increase in ROS could alter the mitochondrial membrane potential [65]. We also demonstrated that BSN could stimulate the mitochondrial membrane potential and cause a GSH/GSSG imbalance. In addition, the levels of ER stress-related proteins ATF4 and CHOP were significantly augmented upon BSN treatment. These results suggested that BSN-induced paraptosis was accompanied by increased ROS production, mitochondrial damage, and ER stress, and cytoplasmic vacuolation.

To decipher the mechanisms implicated in the cell death driven by BSN, the activation status of MAPK was analyzed. The MAPK pathway is well-known for playing a key role in regulating both apoptosis and autophagy [66,67,68]. According to recent studies, the MAPK pathway is also involved in modulating paraptosis. p-JNK and p-ERK in the MAPK pathway have been reported to play a vital role in paraptosis, and the p38 pathway in the MAPK pathway was found to participate in the formation of paraptosis vacuoles [31,32]. Hong et al. found that BSN could inhibit cell proliferation through modulating the MAPK pathway and ROS generation in the hepatocellular carcinoma cell line [45]. We hypothesized that BSN induced apoptosis, autophagy, and paraptosis through stimulating the activation of the MAPK signaling pathway. Our results suggested that BSN could promote the phosphorylation of JNK, ERK, and p38. Additionally, inhibitors of the MAPK signaling pathway could significantly prevent BSN-induced cell death. Next, we confirmed whether these effects of BSN were specific. When Z-DEVD-FMK (apoptosis inhibitor), 3-MA (autophagy inhibitor), and the paraptosis inhibitor (CHX) were treated, respectively, the other two processes were increased. Collectively, BSN triggered three distinct cell death processes (apoptosis, autophagy, and paraptosis) in CML cells through distinct mechanisms. It is still not clear whether the dose of BSN employed in our experiments is relevant to an in vivo situation and hence further studies are needed.

## 5. Conclusions

In conclusion, BSN exerted a substantial impact on the apoptosis, autophagy, and paraptosis cell death process in CML cells. Moreover, these actions were mediated through the stimulating activation of the MAPK signaling pathway. Therefore, BSN might have a potential role in cancer prevention and treatment by affecting multiple cell death processes. However, preclinical studies are needed to further validate these important findings.

## Data Availability

All data are freely available with this article.

## Supplementary Materials

The following supporting information can be downloaded at: https://www.mdpi.com/article/10.3390/biology12020307/s1, Figure S1: Western blot data for Figure 1C; Figure S2: Western blot data for Figure 2A; Figure S3: Western blot data for Figure 2B; Figure S4: Western blot data for Figure 2D; Figure S5: Western blot data for Figure 3D; Figure S6: Western blot data for Figure 4A; Figures S7–S10: Western blot data for Figure 4B; Figure S11: Western blot data for Figure 4C; Figures S12 and S13: Full Western blot images for Figure 1C; Figures S14 and S15: Full Western blot images for Figure 2A; Figures S16 and S17: Full Western blot images for Figure 2B; Figures S18 and S19: Full Western blot images for Figure 2D; Figure S20: Full Western blot images for Figure 3D; Figures S21–S23: Full Western blot images for Figure 4A; Figures S24–S29: Full Western blot images for Figure 4B; Figures S30 and S31: full Western blot images for Figure 4C.
