# Supplementary material for: Brassinin Induces Apoptosis, Autophagy, and Paraptosis via MAPK Signaling Pathway Activation in Chronic Myelogenous Leukemia Cells
Source: Biology (Basel). 2023 Feb 14;12(2):307. doi: 10.3390/biology12020307 (PMC9953140; doi:10.3390/biology12020307)
Supplement: Supplementary file 1 [file biology-12-00307-s001.zip › biology-2097339-supplementary.pdf]

Fig. S1

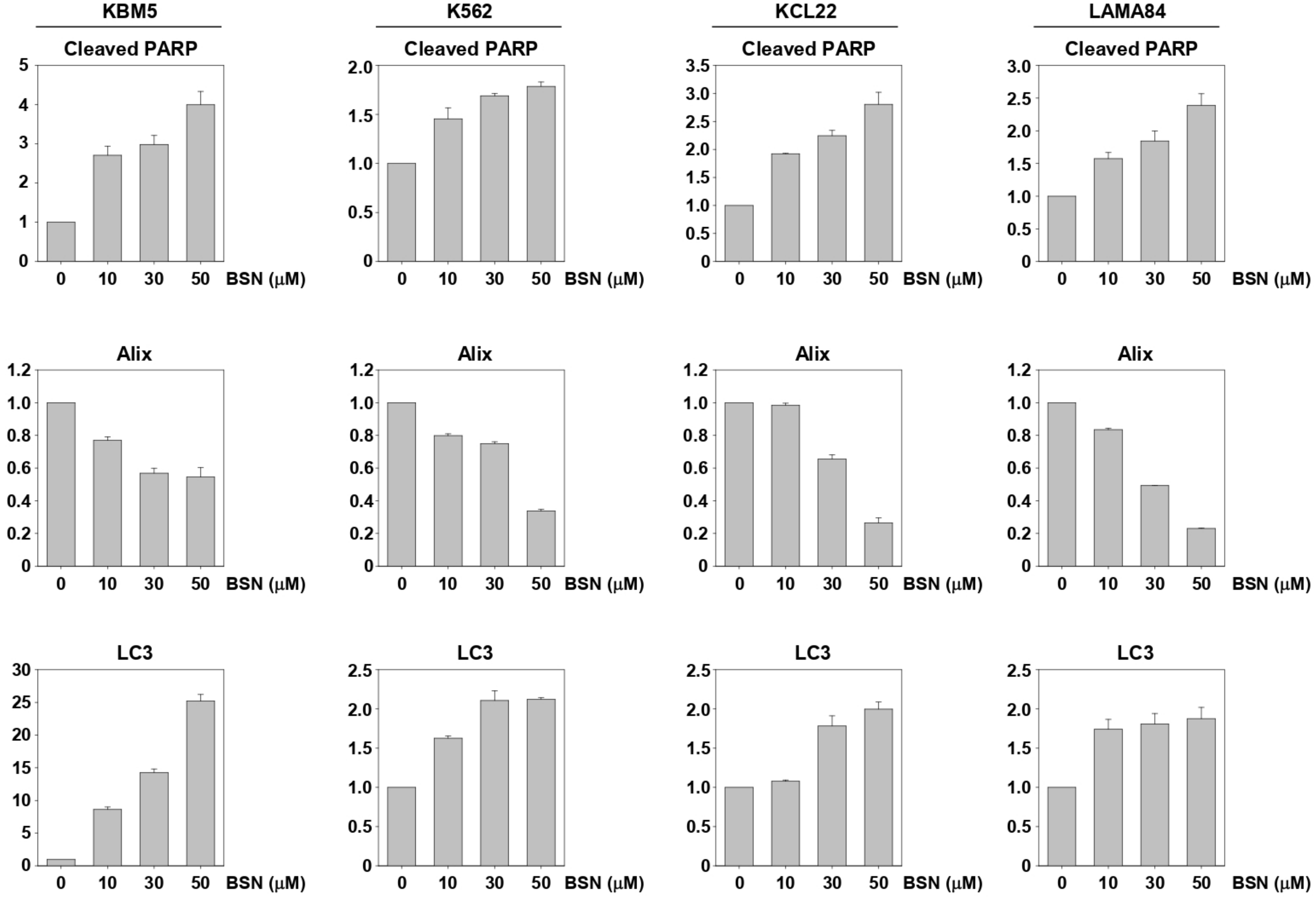

Fig. S2

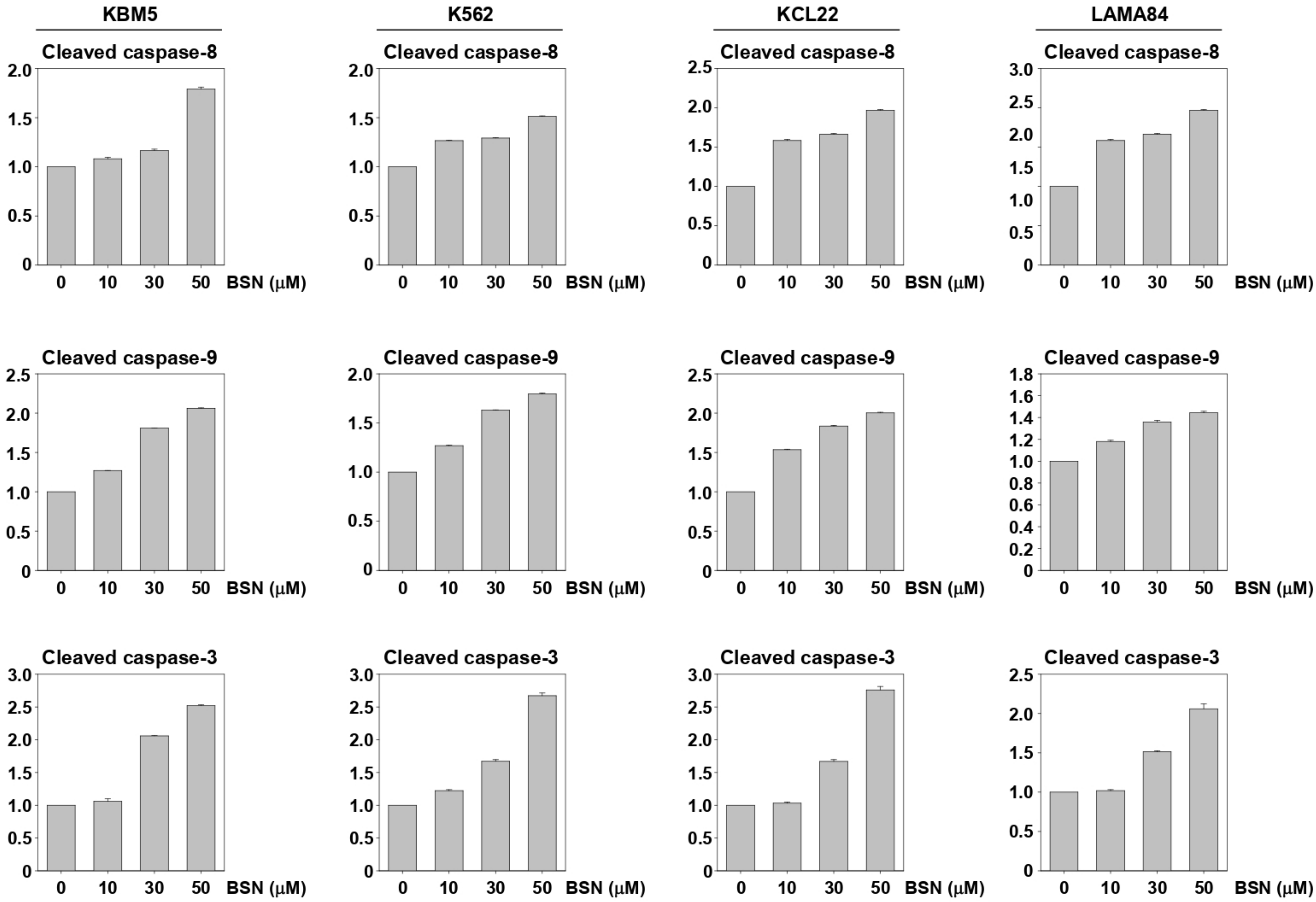
KBM5
K562
KCL22
LAMA84
Cleaved caspase-8
Cleaved caspase-9
Cleaved caspase-3
0
10
30
50
BSN (μM)

Fig.S3

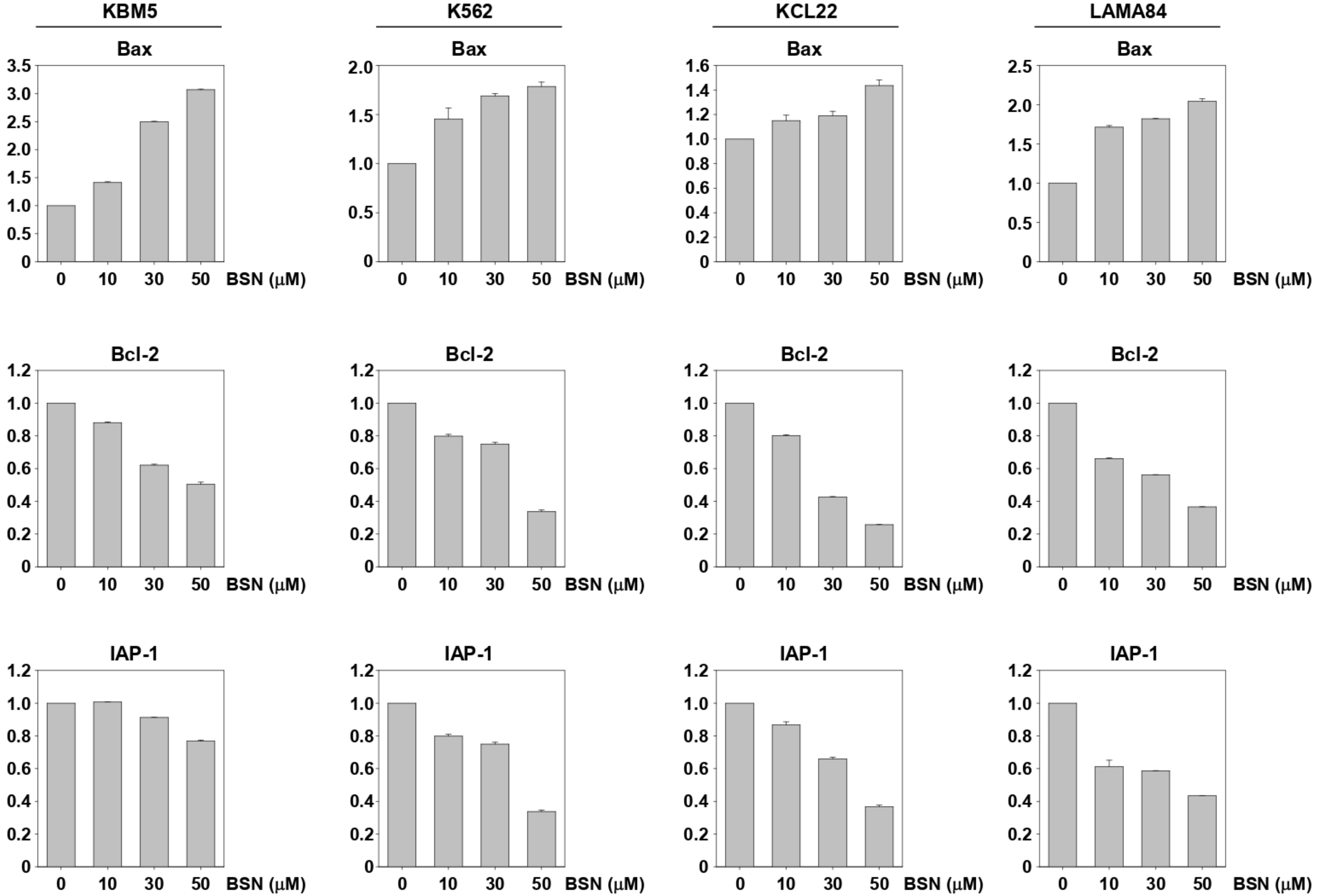

Fig.S4

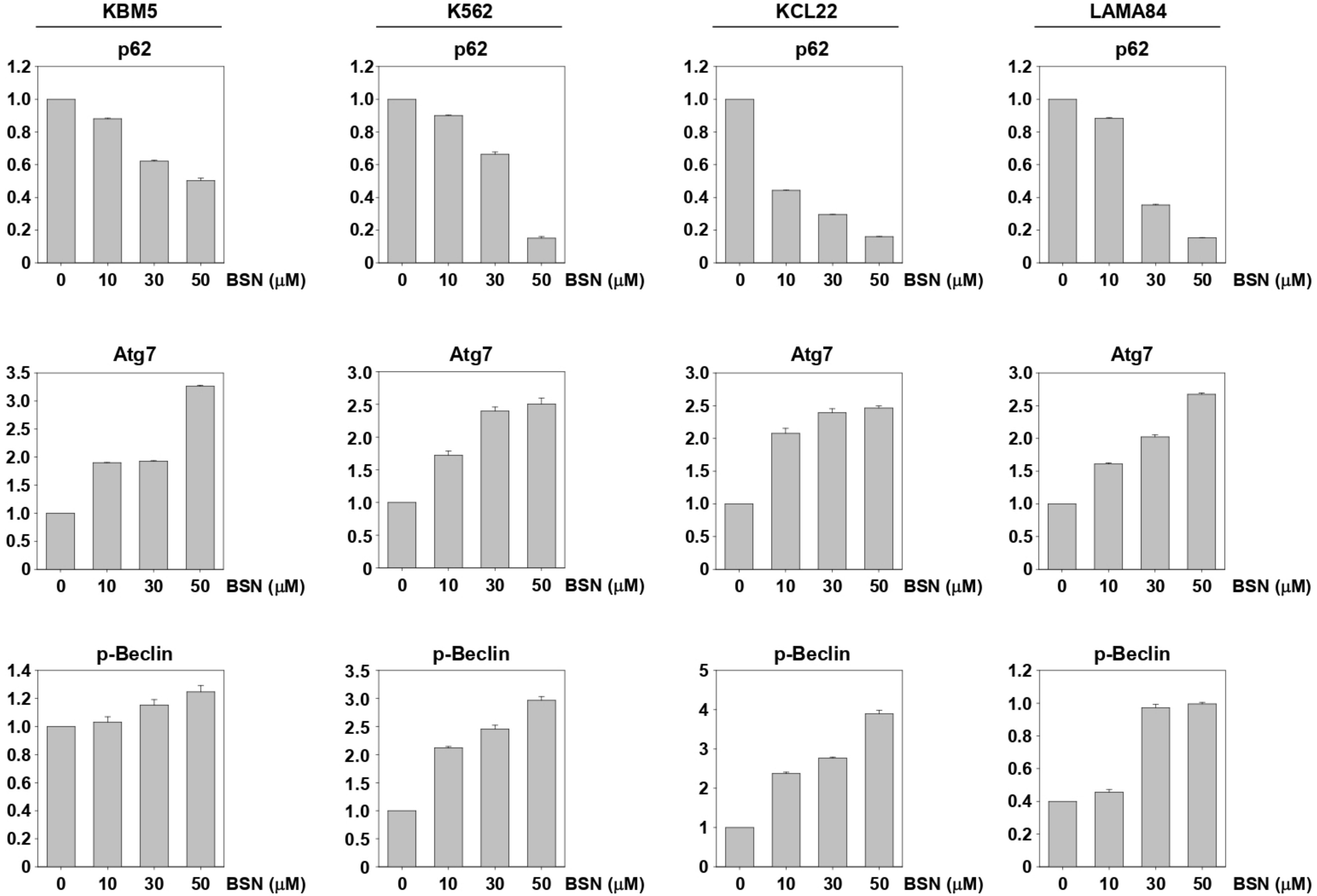

Fig.S5

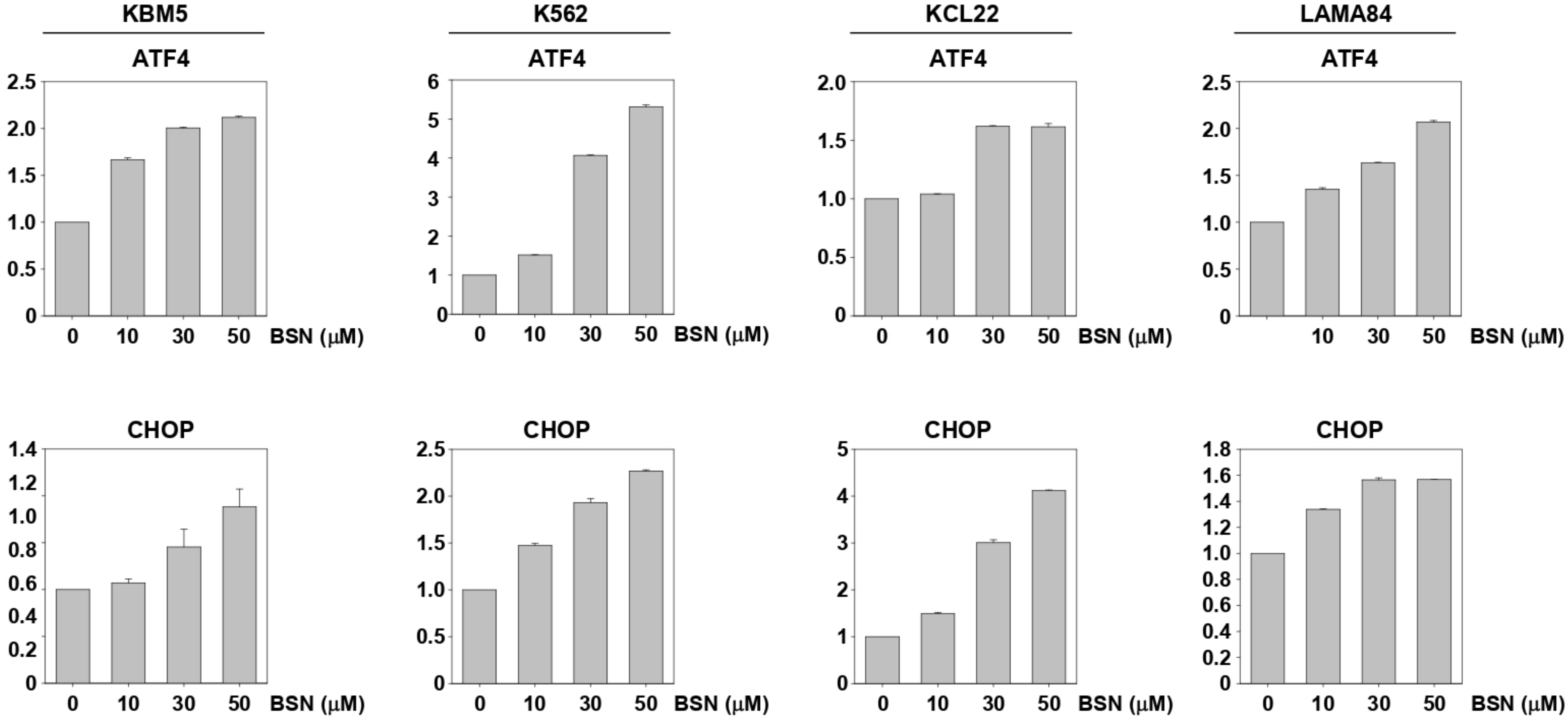
KBM5
K562
KCL22
LAMA84
ATF4
ATF4
ATF4
ATF4
2.5 2.0 1.5 1.0 0.5 0
6 5 4 3 2 1 0
2.0 1.5 1.0 0.5 0
2.5 2.0 1.5 1.0 0.5 0
0 10 30 50 BSN (μM)
0 10 30 50 BSN (μM)
0 10 30 50 BSN (μM)
10 30 50 BSN (μM)
CHOP
CHOP
CHOP
CHOP
1.4 1.2 1.0 0.8 0.6 0.4 0.2 0
2.5 2.0 1.5 1.0 0.5 0
5 4 3 2 1 0
1.8 1.6 1.4 1.2 1.0 0.8 0.6 0.4 0.2 0
0 10 30 50 BSN (μM)
0 10 30 50 BSN (μM)
0 10 30 50 BSN (μM)
0 10 30 50 BSN (μM)

Fig. S6

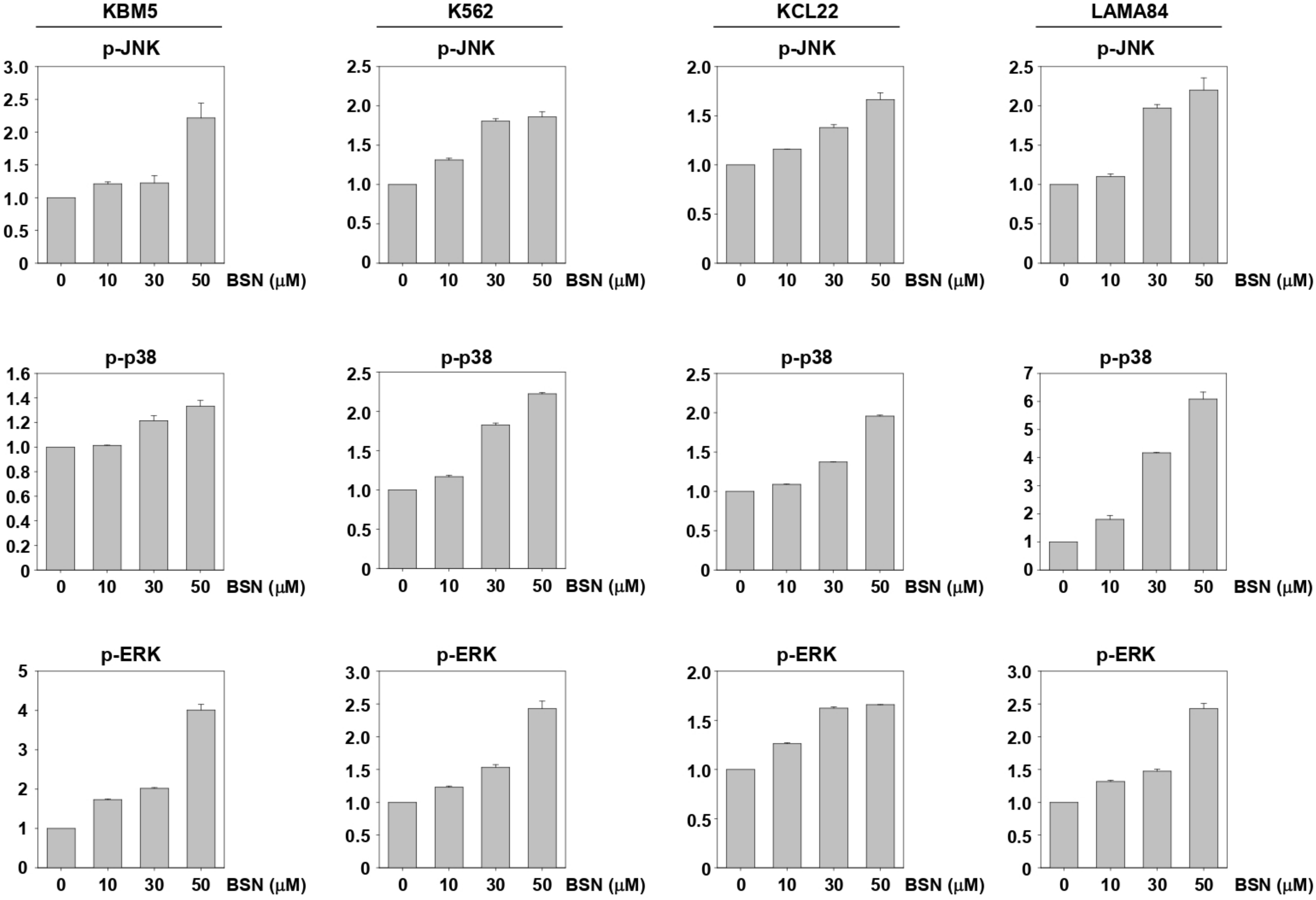

Fig.S7

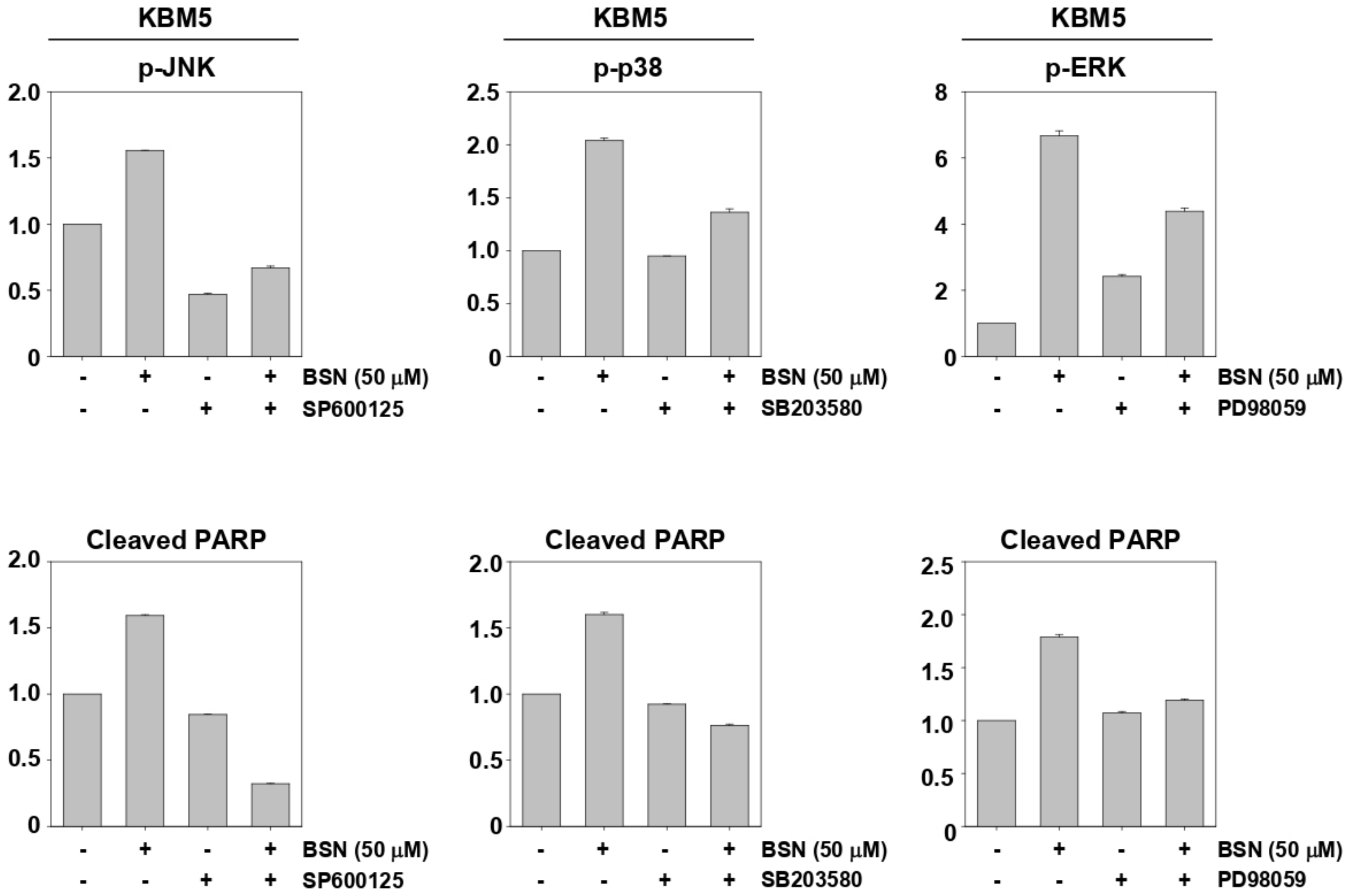
KBM5
p-JNK
2.0
1.5
1.0
0.5
0
- + - + BSN (50 μM)
- - + + SP600125
KBM5
p-p38
2.5
2.0
1.5
1.0
0.5
0
- + - + BSN (50 μM)
- - + + SB203580
KBM5
p-ERK
8
6
4
2
0
- + - + BSN (50 μM)
- - + + PD98059
Cleaved PARP
2.0
1.5
1.0
0.5
0
- + - + BSN (50 μM)
- - + + SP600125
Cleaved PARP
2.0
1.5
1.0
0.5
0
- + - + BSN (50 μM)
- - + + SB203580
Cleaved PARP
2.5
2.0
1.5
1.0
0.5
0
- + - + BSN (50 μM)
- - + + PD98059

Fig. S8

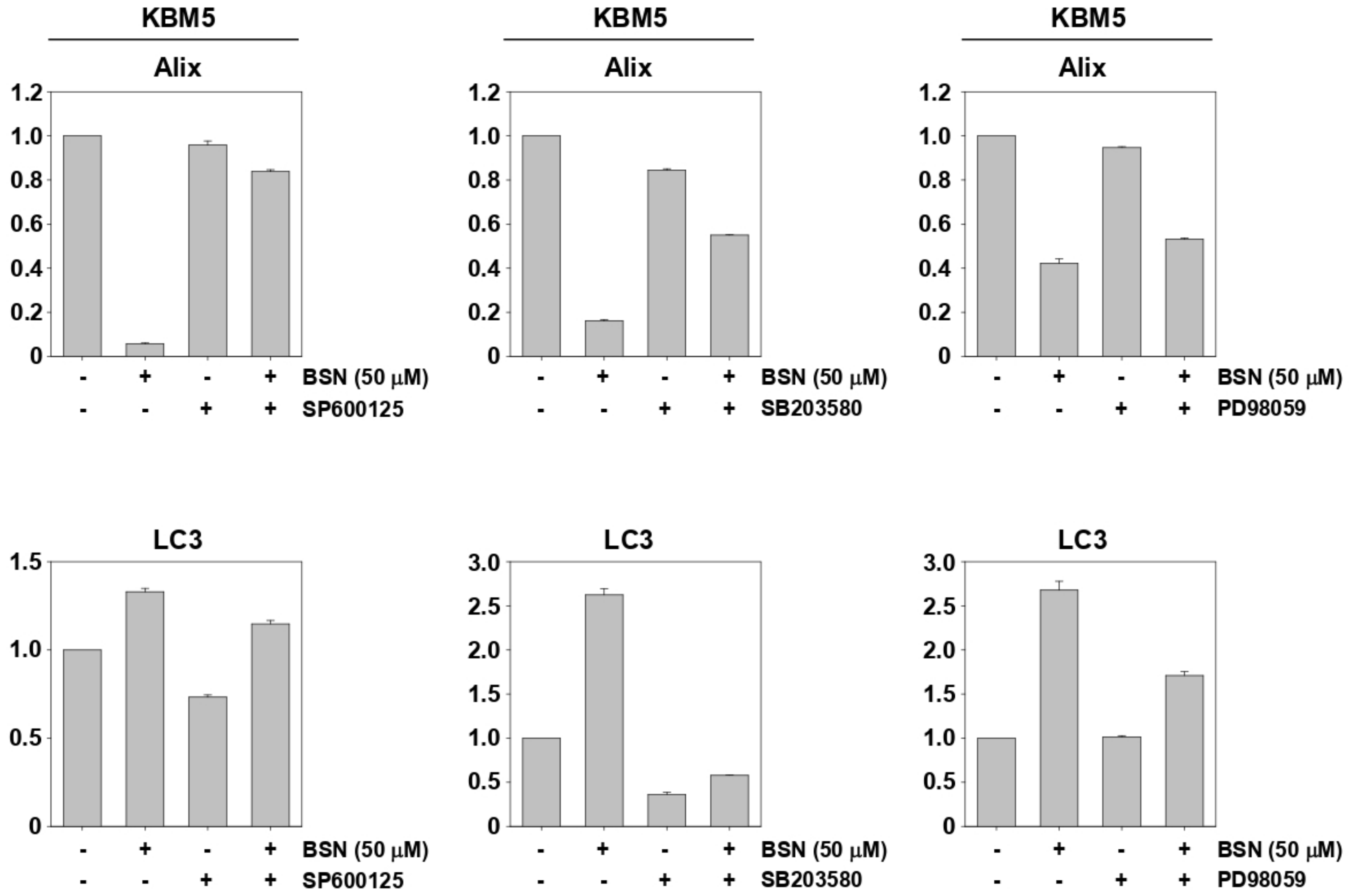
KBM5
Alix
1.2
1.0
0.8
0.6
0.4
0.2
0
- + - + BSN (50 μM)
- - + + SP600125
KBM5
Alix
- + - + BSN (50 μM)
- - + + SB203580
KBM5
Alix
- + - + BSN (50 μM)
- - + + PD98059
LC3
1.5
1.0
0.5
0
- + - + BSN (50 μM)
- - + + SP600125
LC3
3.0
2.5
2.0
1.5
1.0
0.5
0
- + - + BSN (50 μM)
- - + + SB203580
LC3
- + - + BSN (50 μM)
- - + + PD98059

Fig.S9

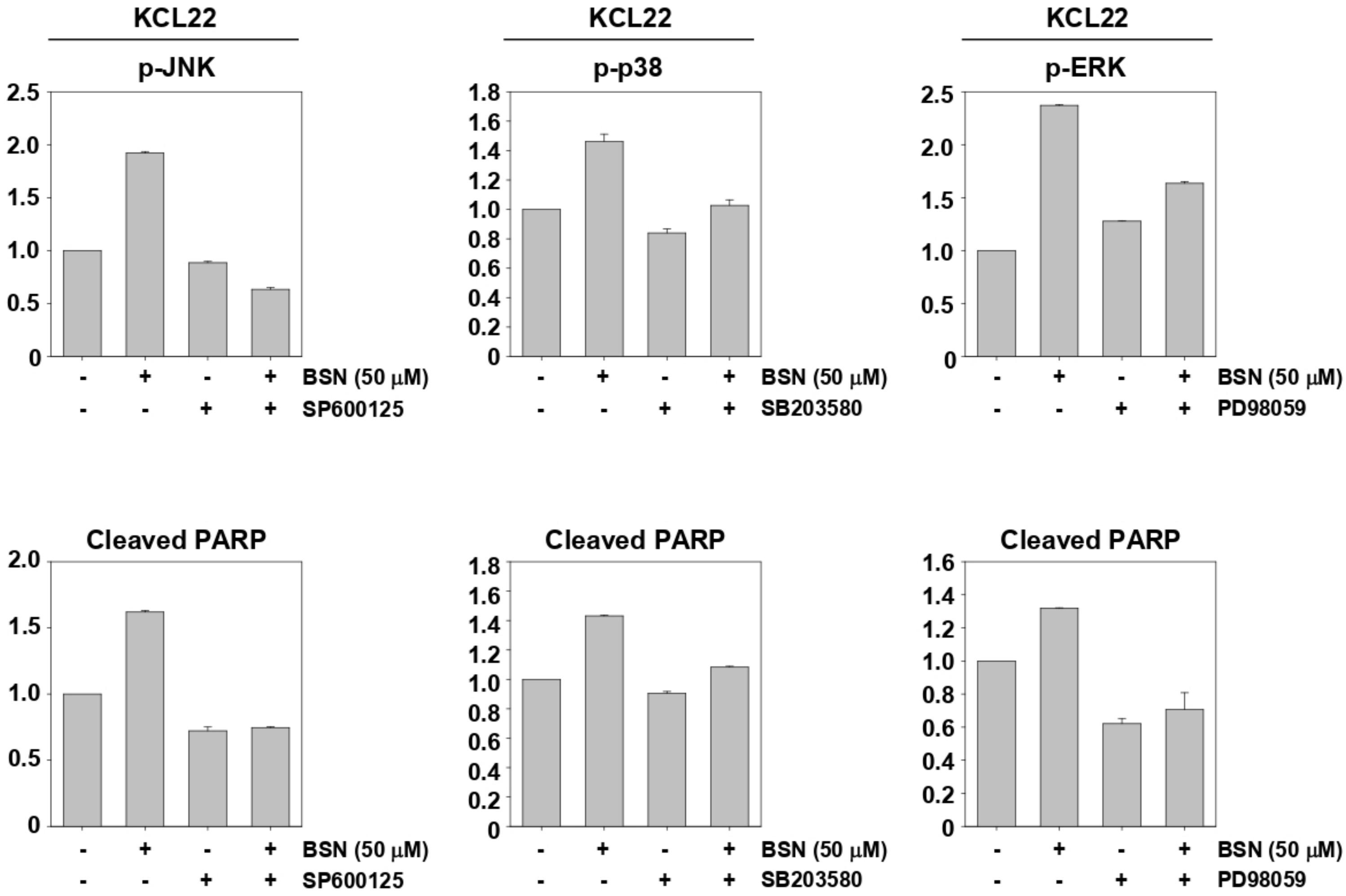
KCL22
p-JNK
BSN (50 μM)
SP600125
KCL22
p-p38
BSN (50 μM)
SB203580
KCL22
p-ERK
BSN (50 μM)
PD98059
Cleaved PARP
BSN (50 μM)
SP600125
Cleaved PARP
BSN (50 μM)
SB203580
Cleaved PARP
BSN (50 μM)
PD98059

Fig. S10

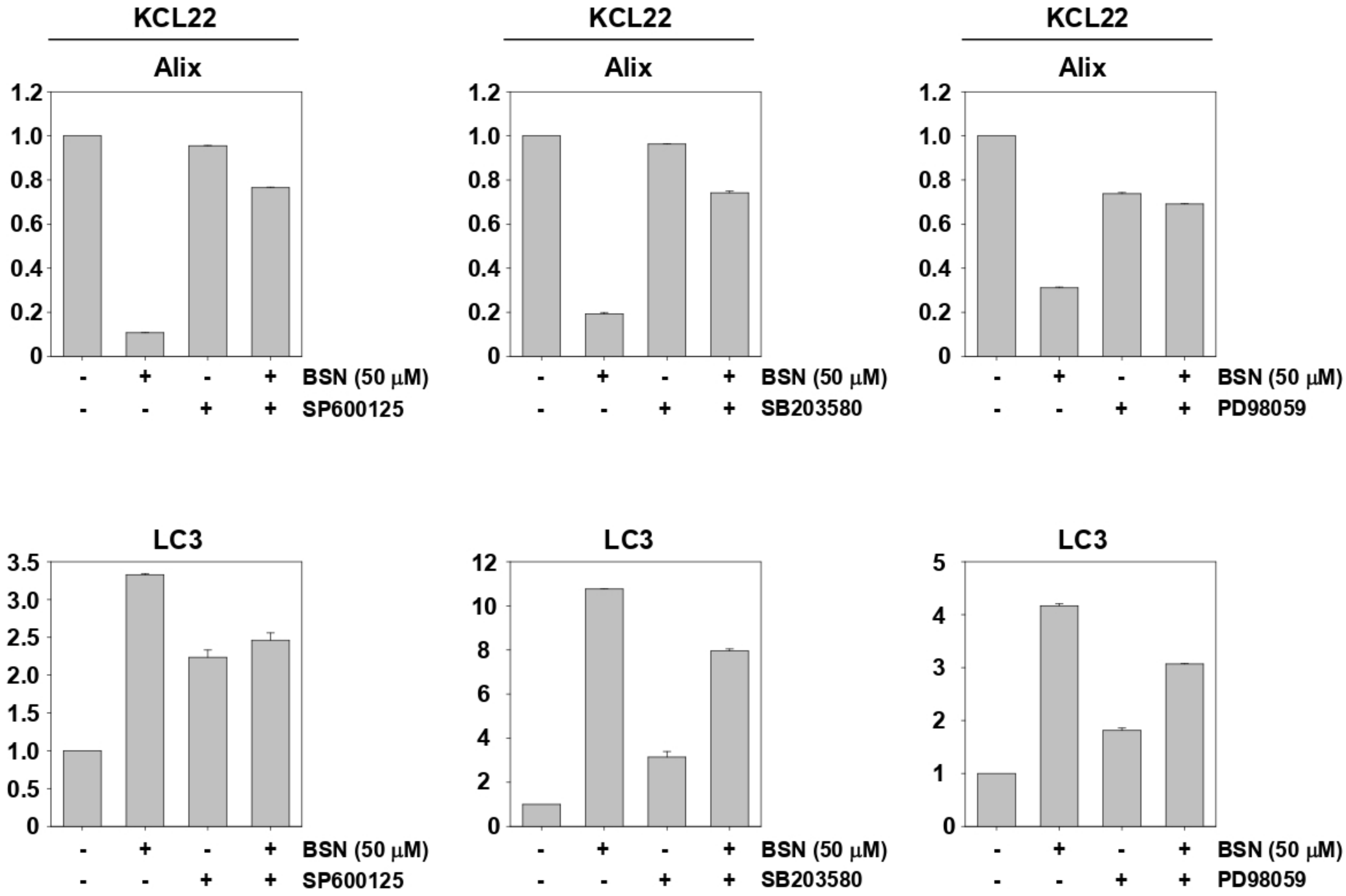
KCL22
Alix
1.2
1.0
0.8
0.6
0.4
0.2
0
- + - + BSN (50 μM)
- - + + SP600125
KCL22
Alix
- + - + BSN (50 μM)
- - + + SB203580
KCL22
Alix
- + - + BSN (50 μM)
- - + + PD98059
LC3
3.5
3.0
2.5
2.0
1.5
1.0
0.5
0
- + - + BSN (50 μM)
- - + + SP600125
LC3
12
10
8
6
4
2
0
- + - + BSN (50 μM)
- - + + SB203580
LC3
5
4
3
2
1
0
- + - + BSN (50 μM)
- - + + PD98059

Fig. S11

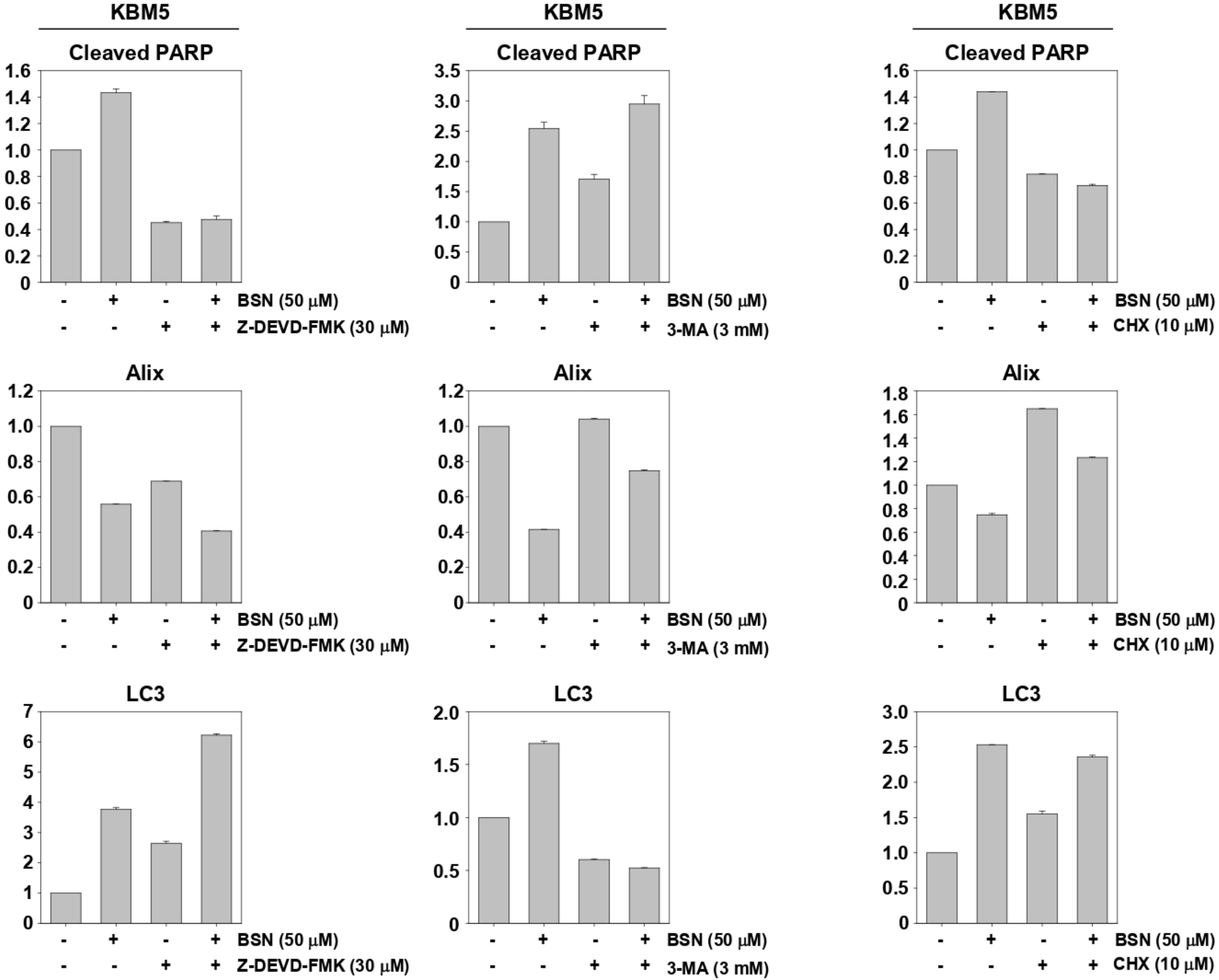
KBM5
Cleaved PARP
Alix
LC3
BSN (50 μM)
Z-DEVD-FMK (30 μM)
3-MA (3 mM)
CHX (10 μM)

Fig.S12

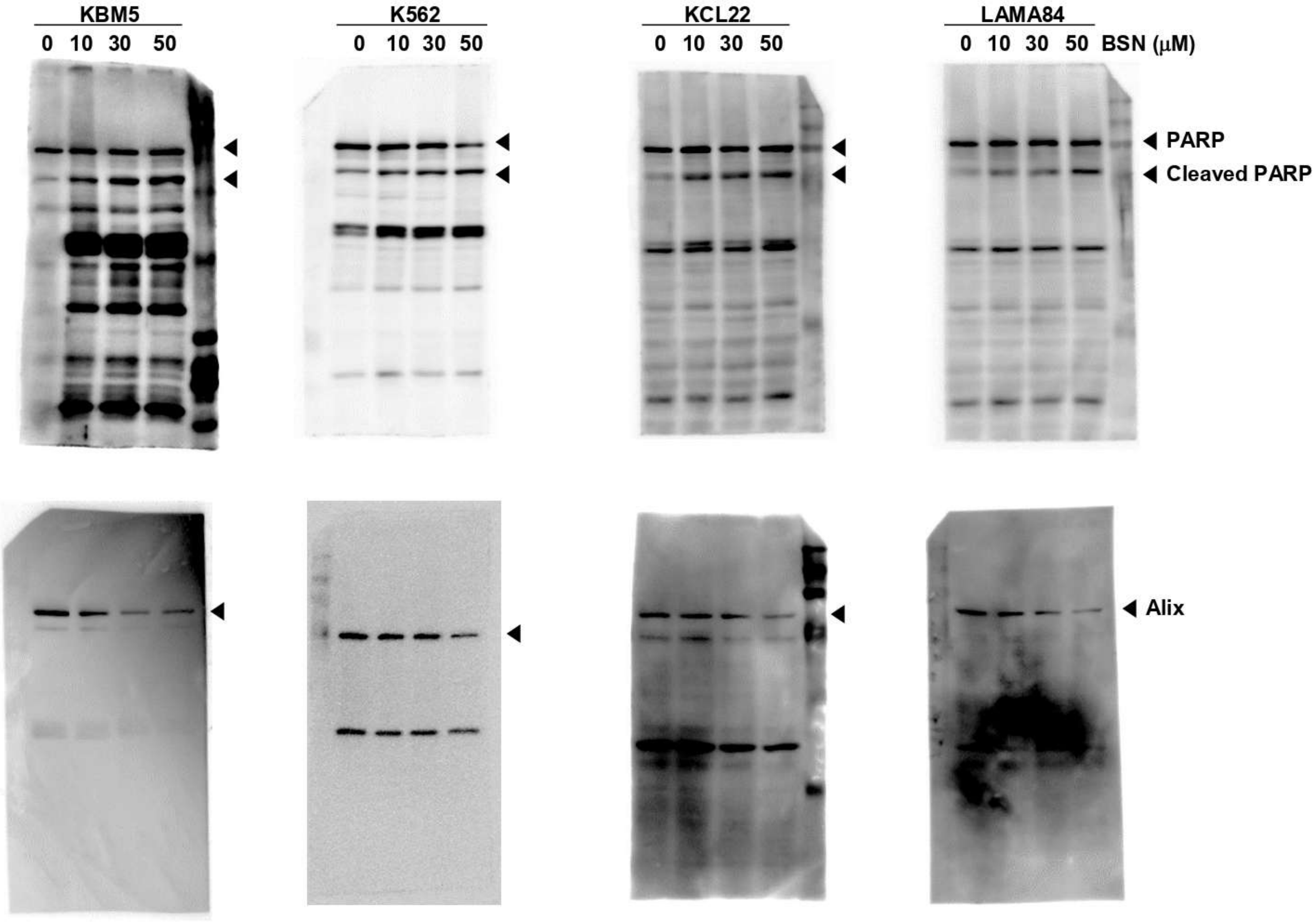

KBM5
K562
KCL22
LAMA84
0 10 30 50 BSN (μM)
PARP
Cleaved PARP
Alix

Fig. S13

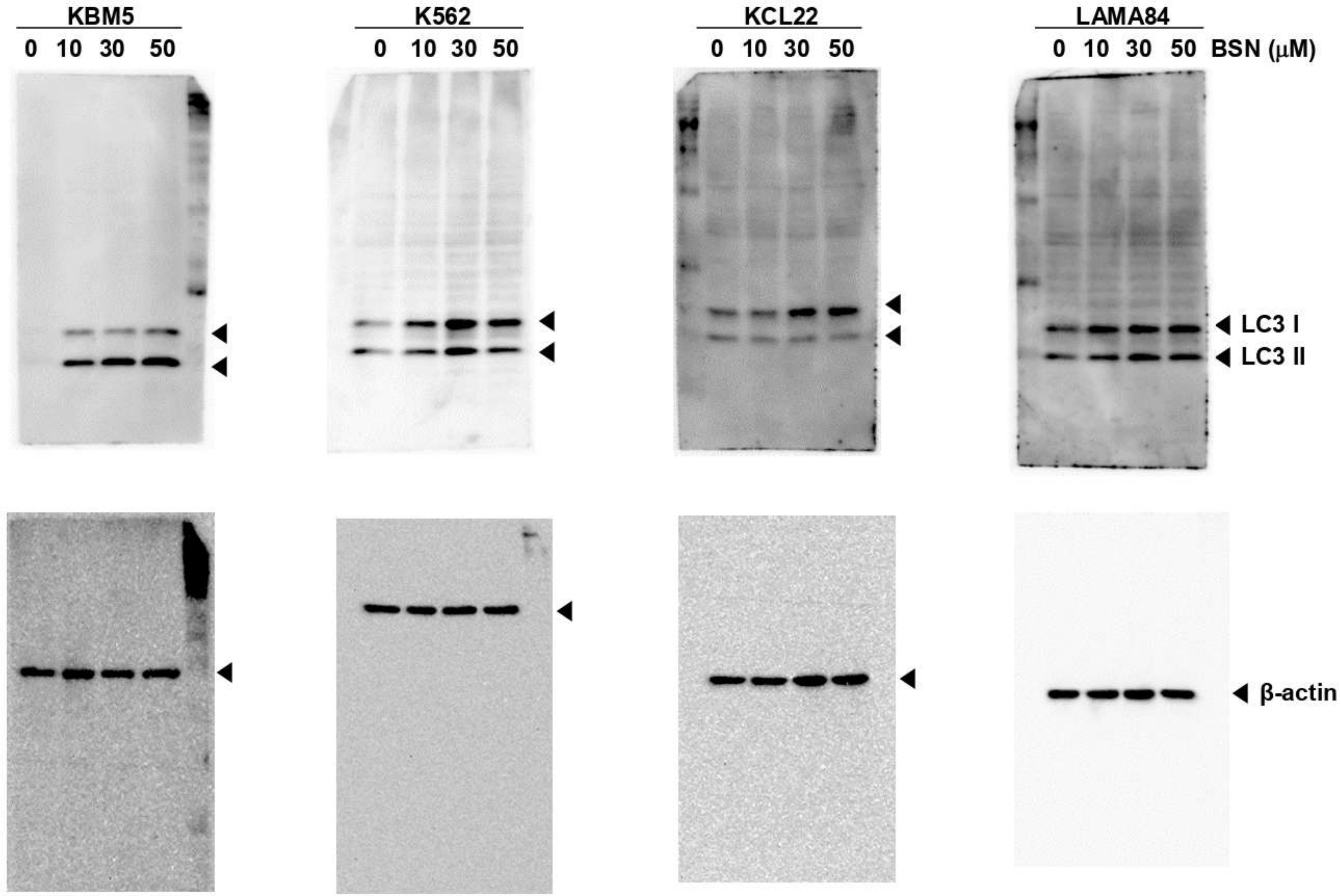
KBM5
K562
KCL22
LAMA84
0 10 30 50
0 10 30 50
0 10 30 50
0 10 30 50 BSN (μM)
LC3 I
LC3 II
β-actin

Fig.S14

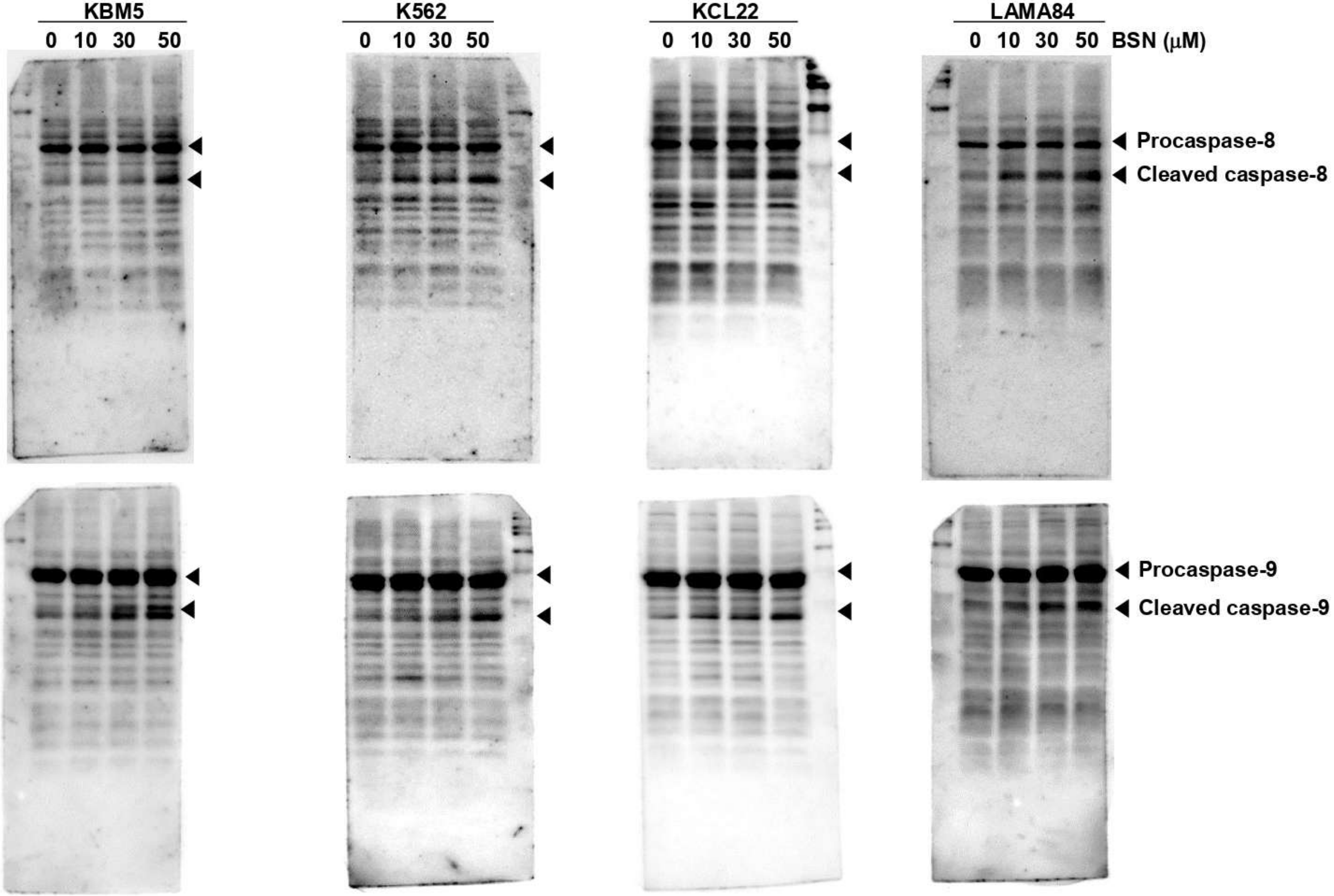
KBM5
K562
KCL22
LAMA84
0 10 30 50
BSN (μM)
Procaspase-8
Cleaved caspase-8
Procaspase-9
Cleaved caspase-9

Fig.S15

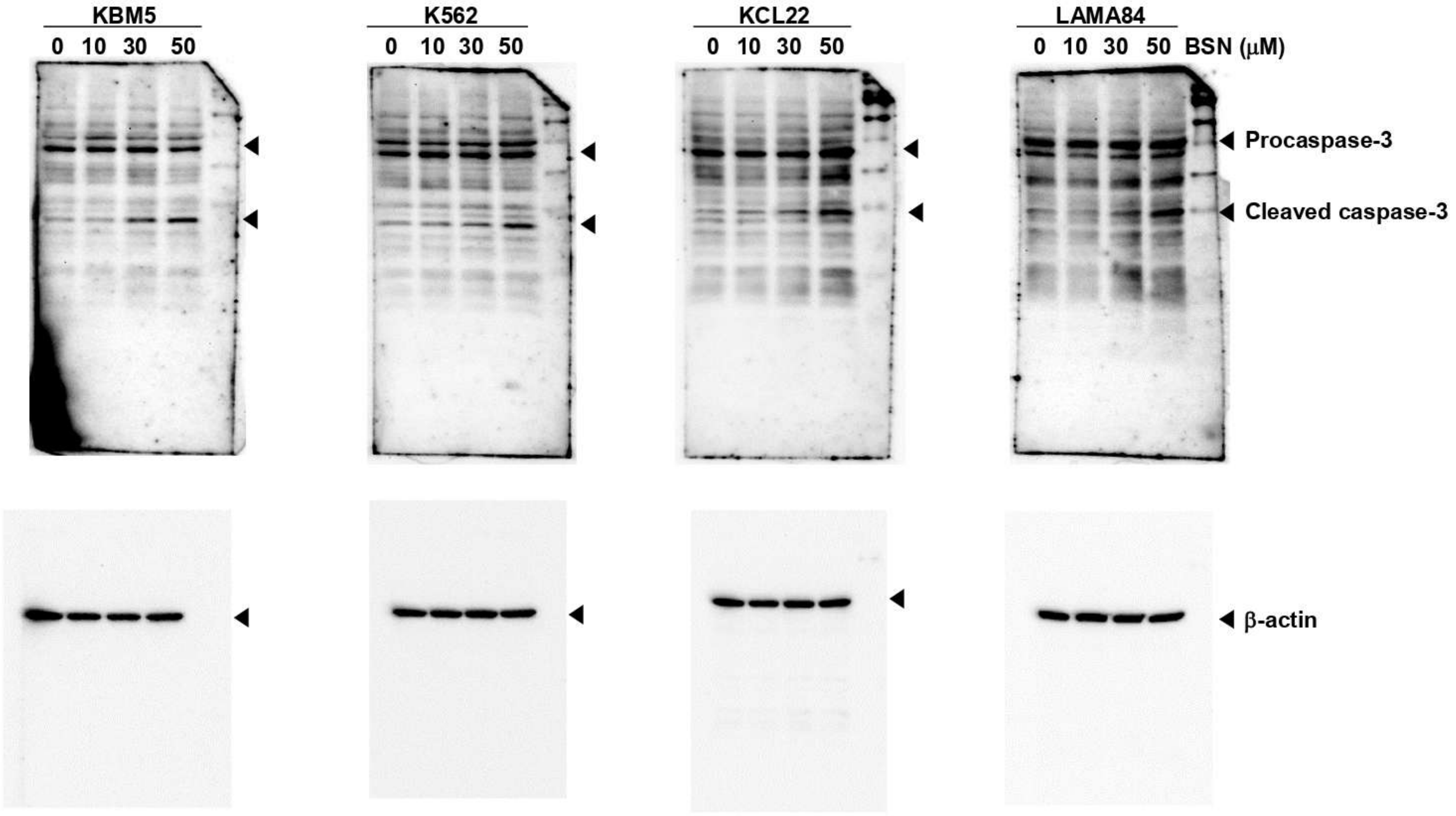

KBM5
K562
KCL22
LAMA84
0 10 30 50
0 10 30 50
0 10 30 50
0 10 30 50 BSN (μM)
Procaspase-3
Cleaved caspase-3
β-actin

Fig.S16

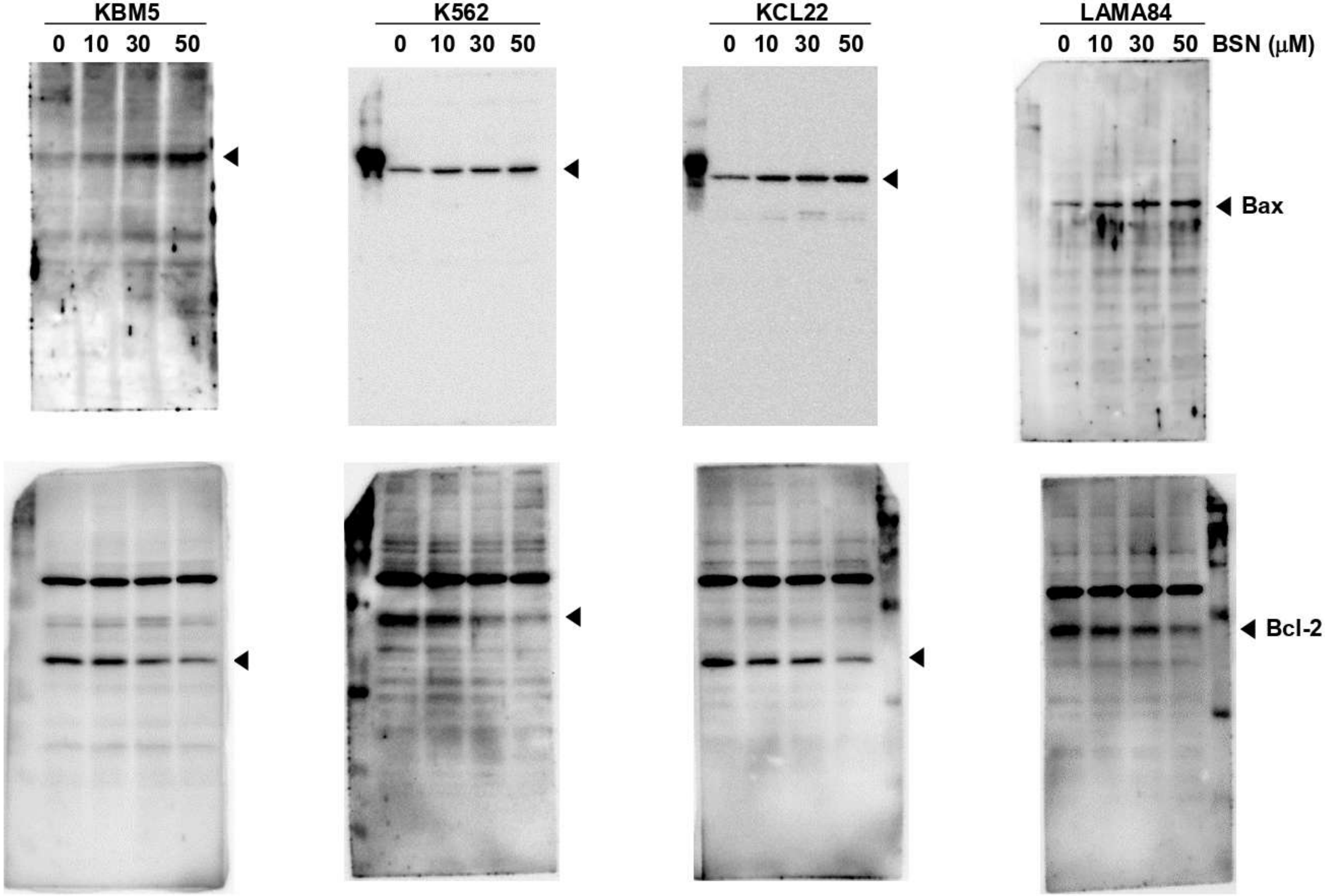

KBM5
K562
KCL22
LAMA84
0 10 30 50
0 10 30 50
0 10 30 50
0 10 30 50 BSN (μM)
Bax
Bcl-2

Fig.S17

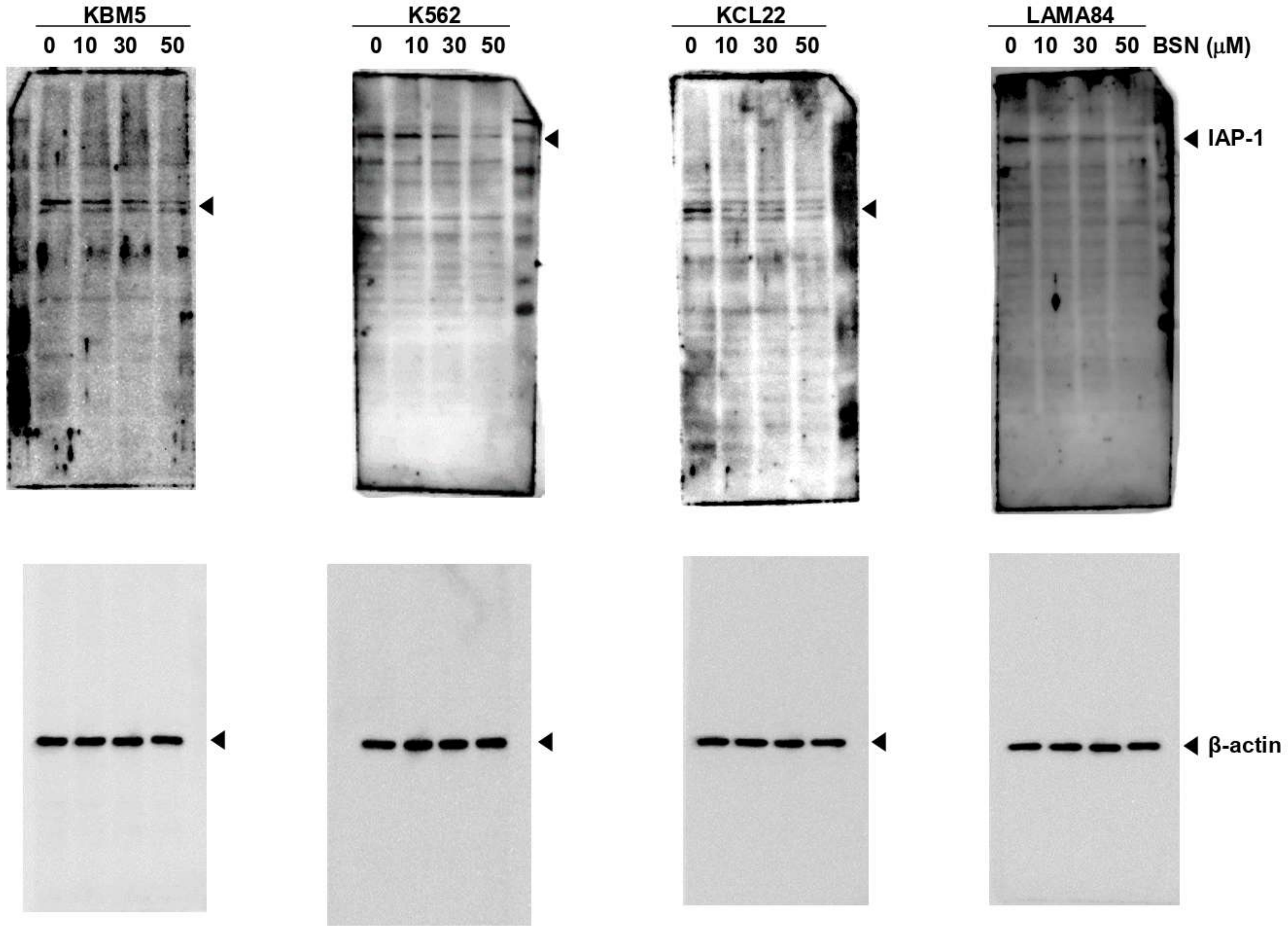
KBM5
0 10 30 50
K562
0 10 30 50
KCL22
0 10 30 50
LAMA84
0 10 30 50 BSN (μM)
IAP-1
β-actin

Fig.S18

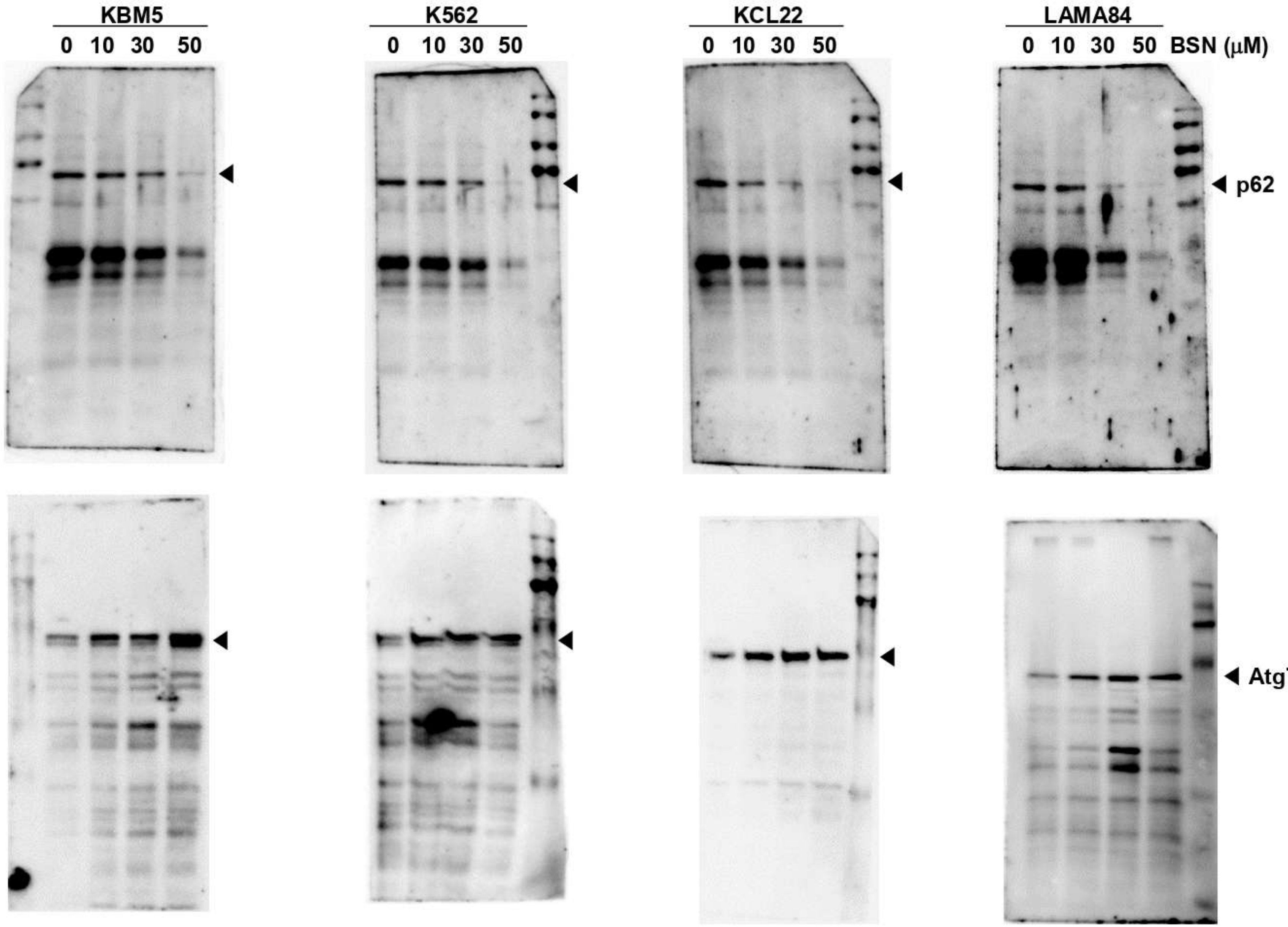

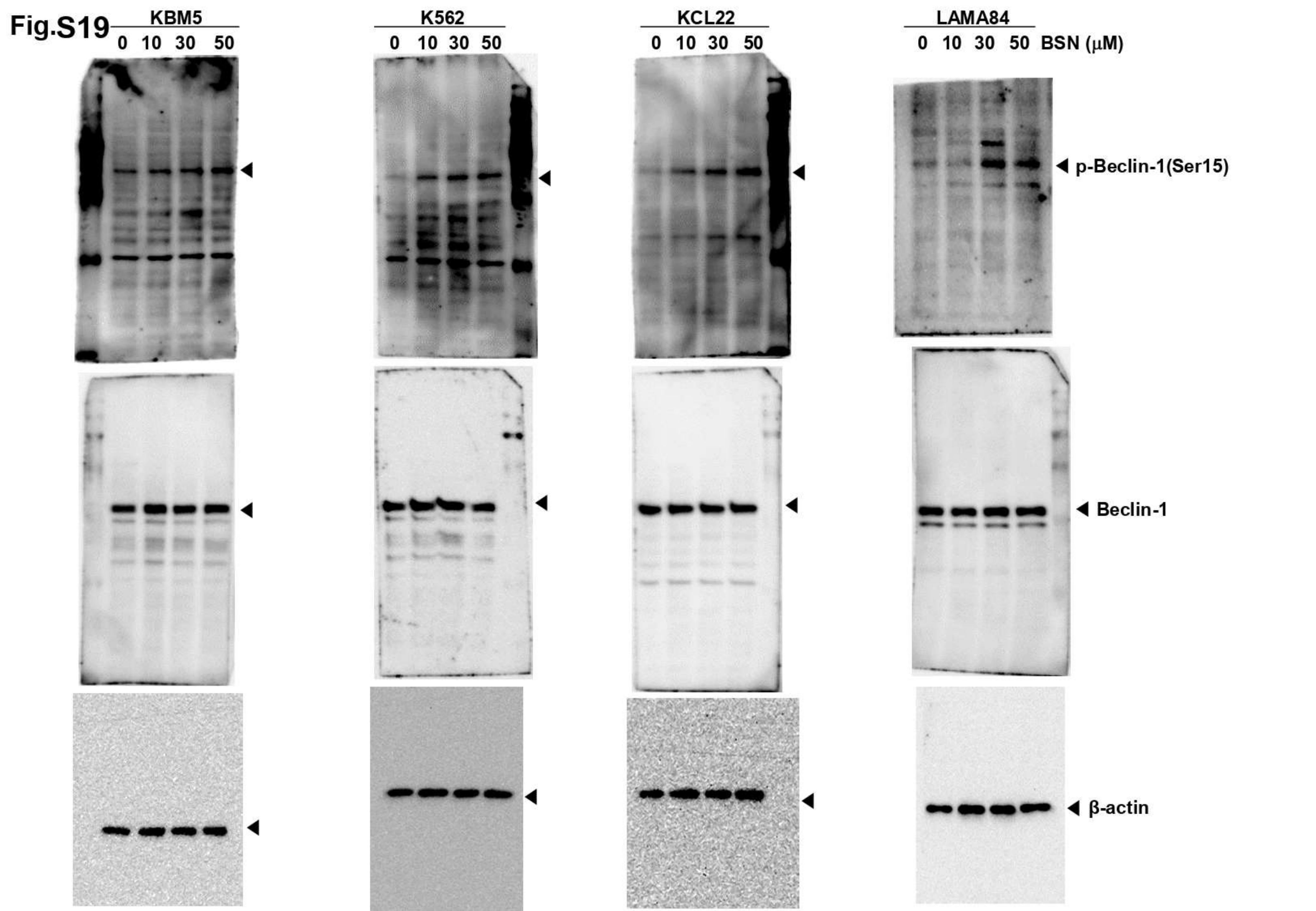
Fig.S19
KBM5
K562
KCL22
LAMA84
0 10 30 50
0 10 30 50
0 10 30 50
0 10 30 50 BSN (μM)
p-Beclin-1(Ser15)
Beclin-1
β-actin

Fig.S20

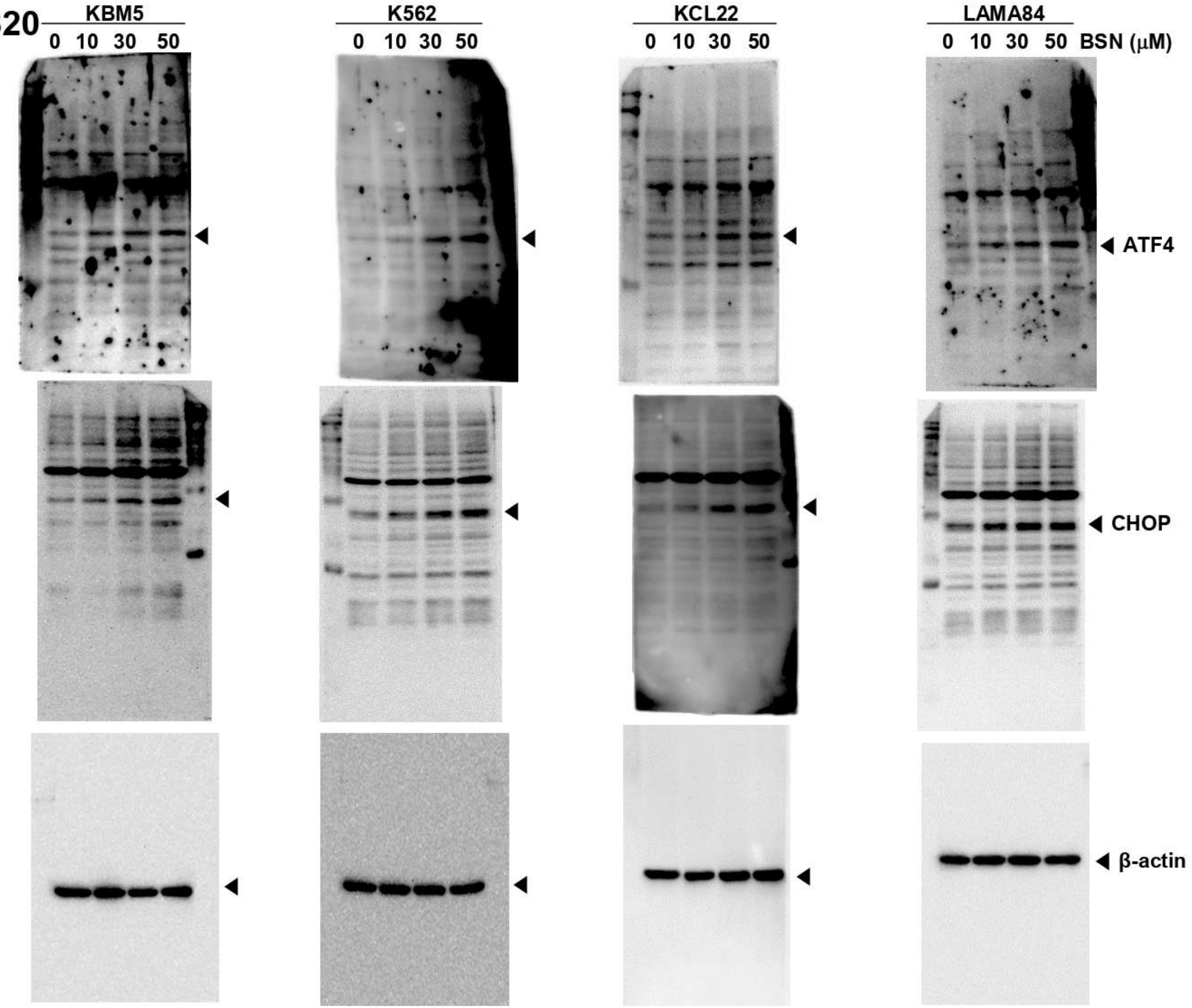

KBM5
K562
KCL22
LAMA84
0 10 30 50
0 10 30 50
0 10 30 50
0 10 30 50 BSN (μM)
ATF4
CHOP
β-actin

Fig. S21

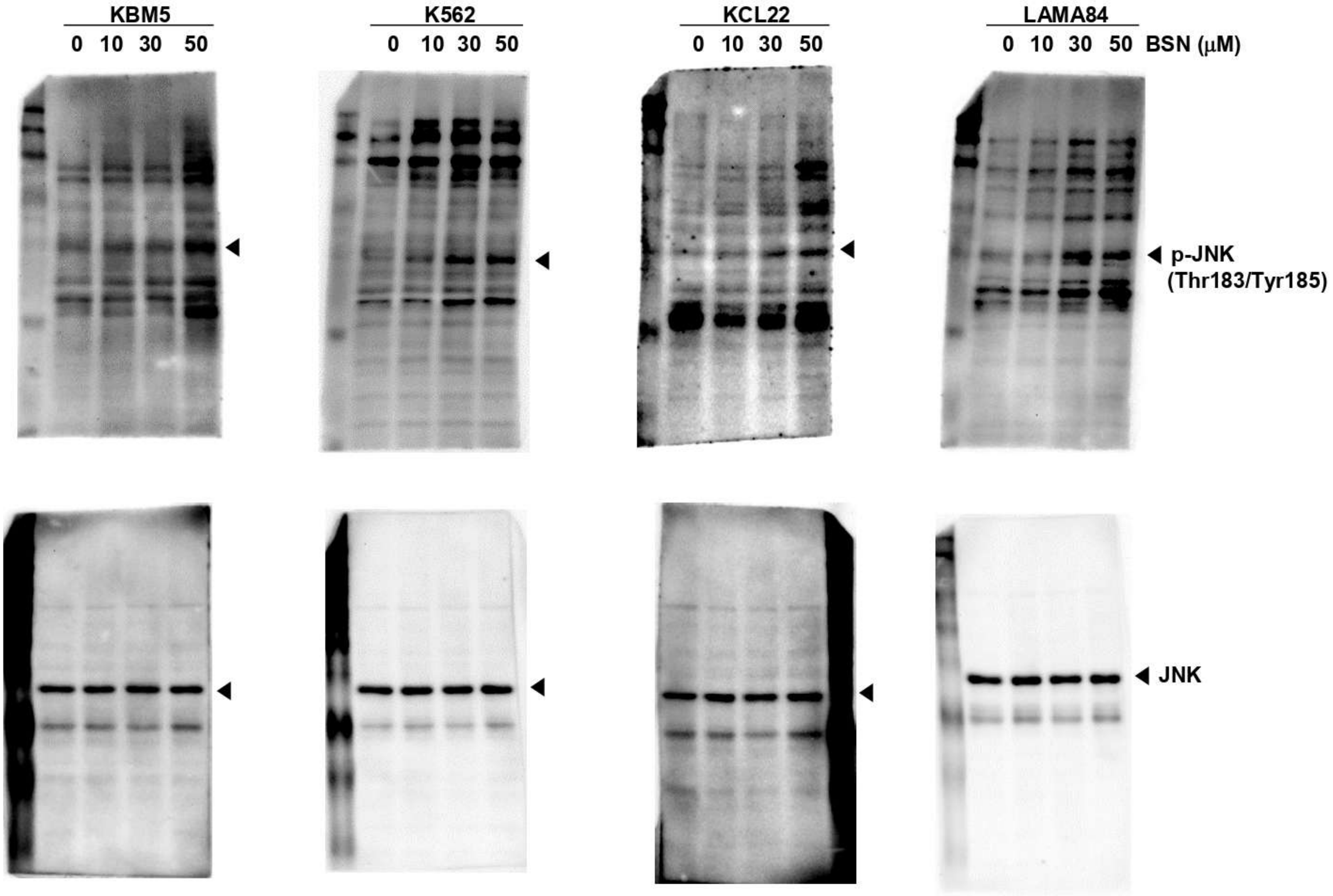
KBM5
K562
KCL22
LAMA84
0 10 30 50
BSN (μM)
p-JNK
(Thr183/Tyr185)
JNK

Fig.S22

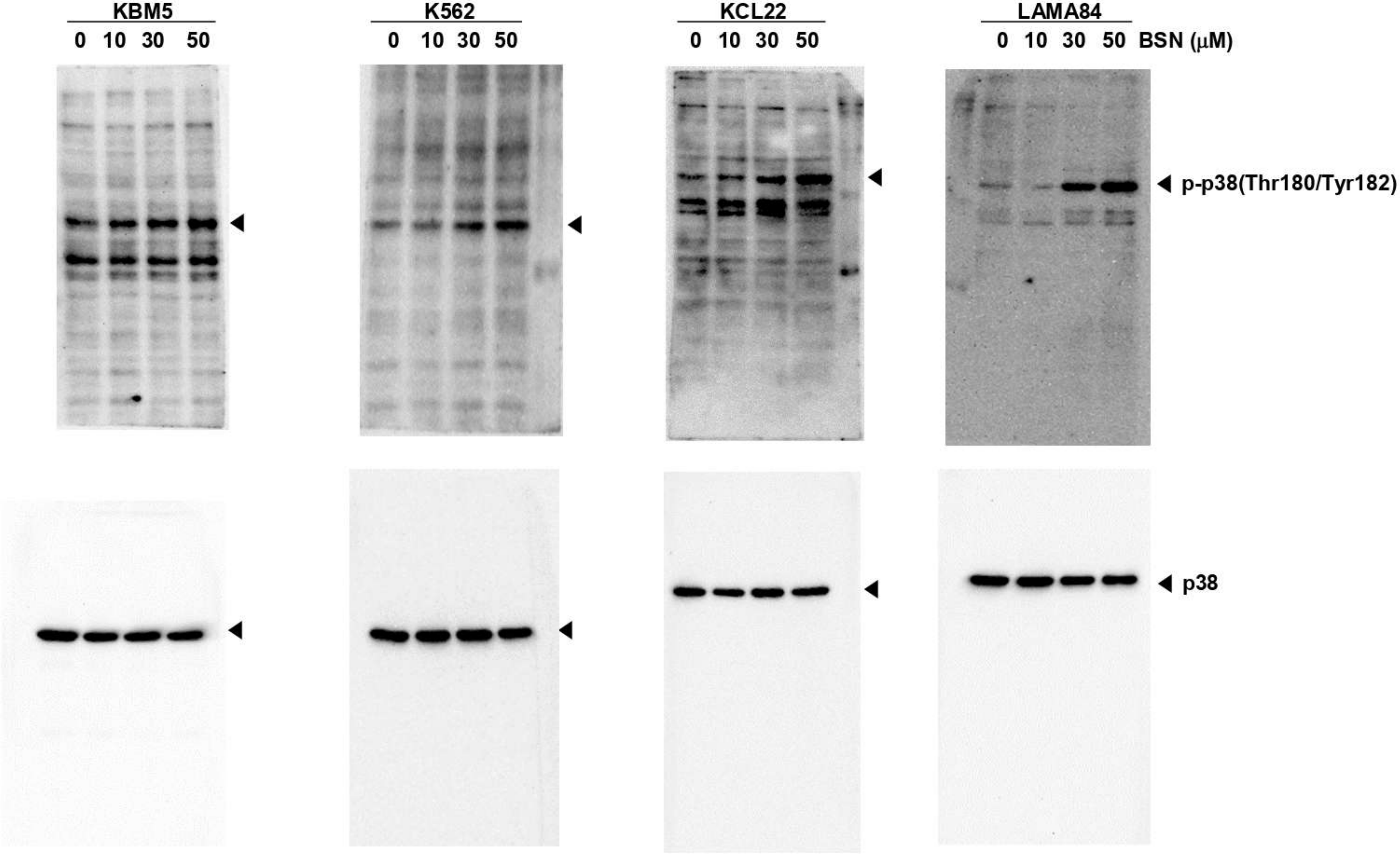

KBM5
K562
KCL22
LAMA84
0 10 30 50
0 10 30 50
0 10 30 50
0 10 30 50 BSN (μM)
p-p38(Thr180/Tyr182)
p38

Fig. S23

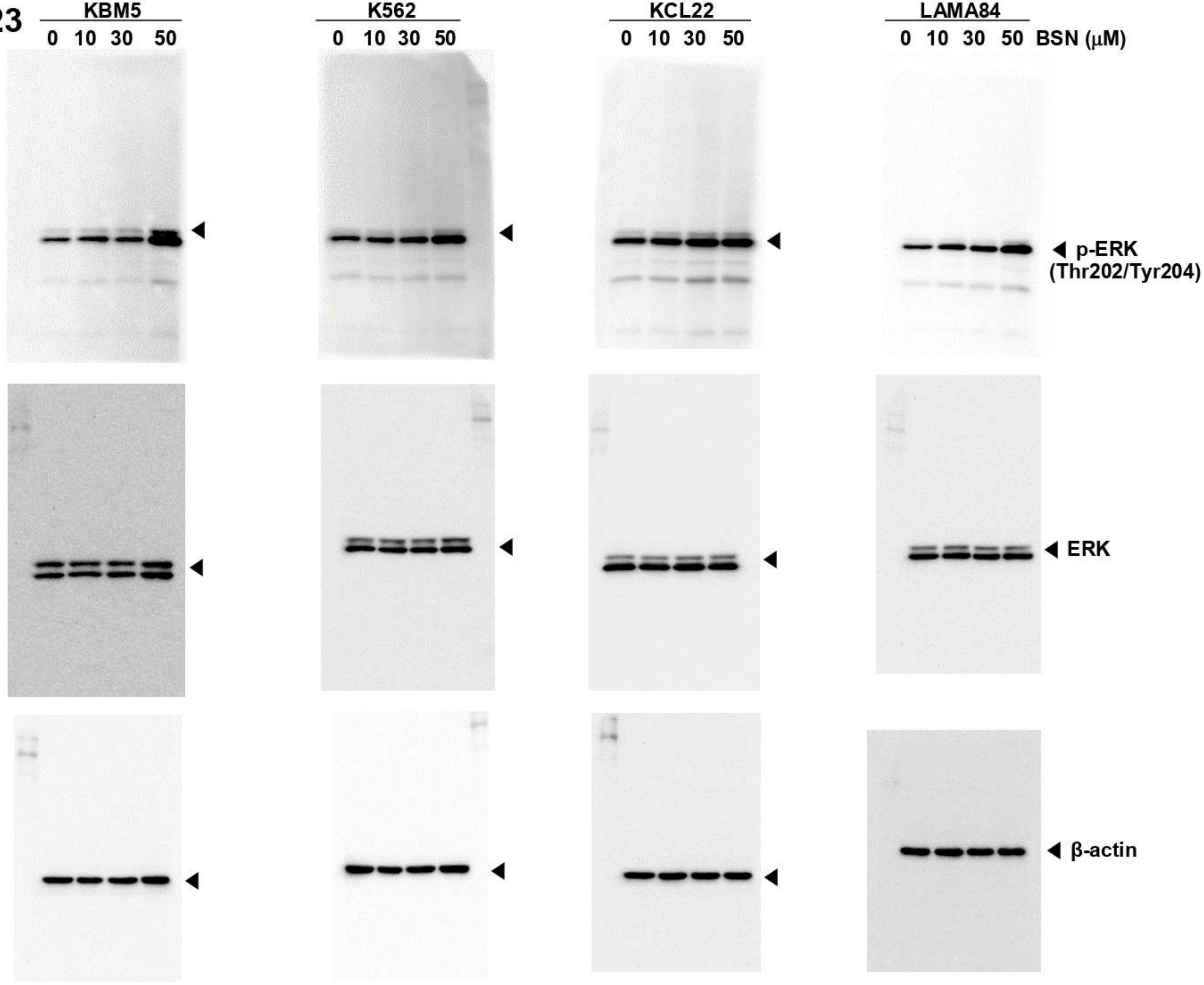

KBM5
K562
KCL22
LAMA84
0 10 30 50
0 10 30 50
0 10 30 50
0 10 30 50 BSN (μM)
p-ERK (Thr202/Tyr204)
ERK
β-actin

Fig. S24

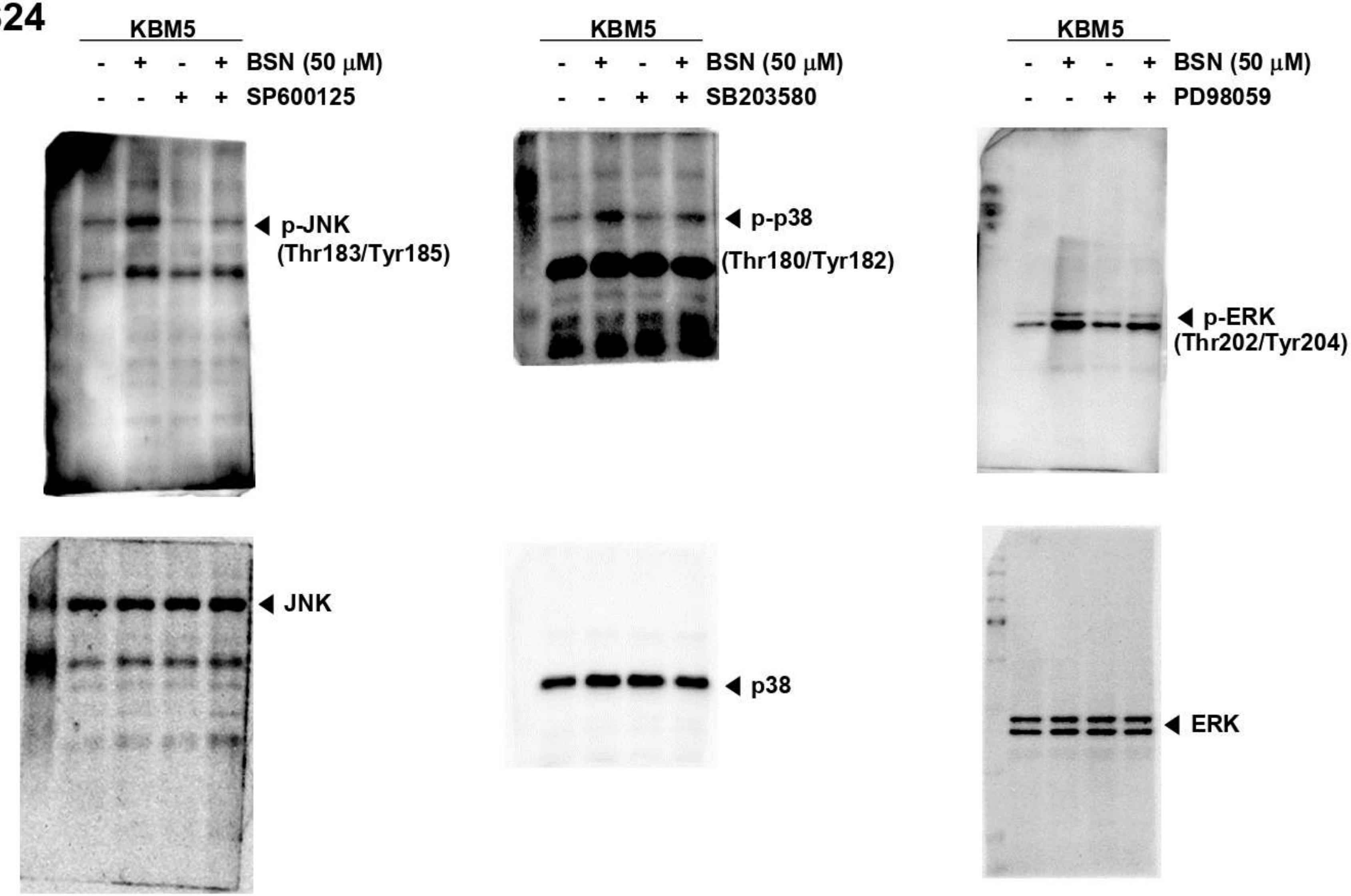
KBM5
- + - + BSN (50 μM)
- - + + SP600125
p-JNK (Thr183/Tyr185)
JNK
KBM5
- + - + BSN (50 μM)
- - + + SB203580
p-p38 (Thr180/Tyr182)
p38
KBM5
- + - + BSN (50 μM)
- - + + PD98059
p-ERK (Thr202/Tyr204)
ERK

Fig.S25

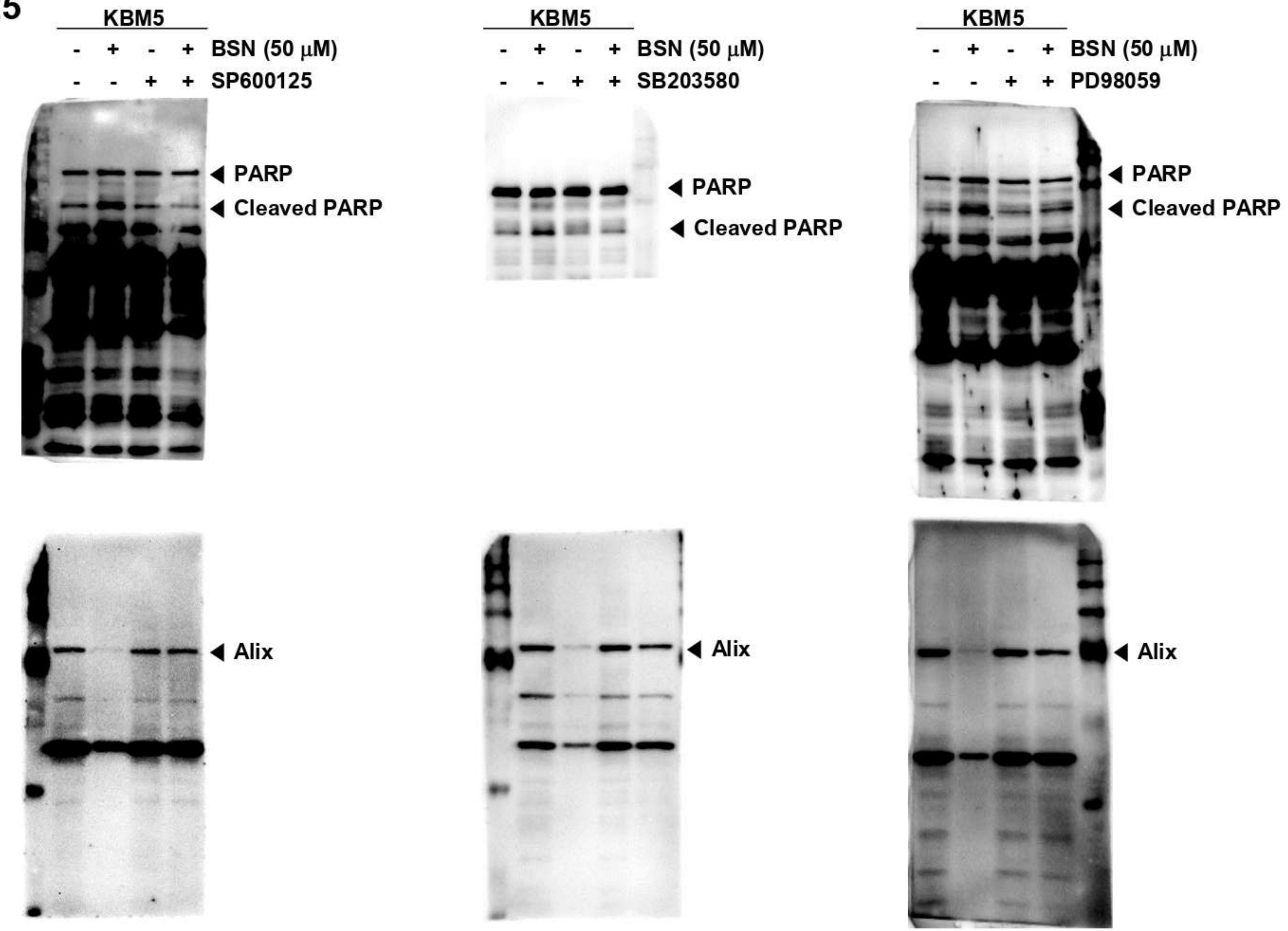

KBM5
- + - + BSN (50 μM)
- - + + SP600125
PARP
Cleaved PARP
Alix
KBM5
- + - + BSN (50 μM)
- - + + SB203580
PARP
Cleaved PARP
Alix
KBM5
- + - + BSN (50 μM)
- - + + PD98059
PARP
Cleaved PARP
Alix

Fig.S26

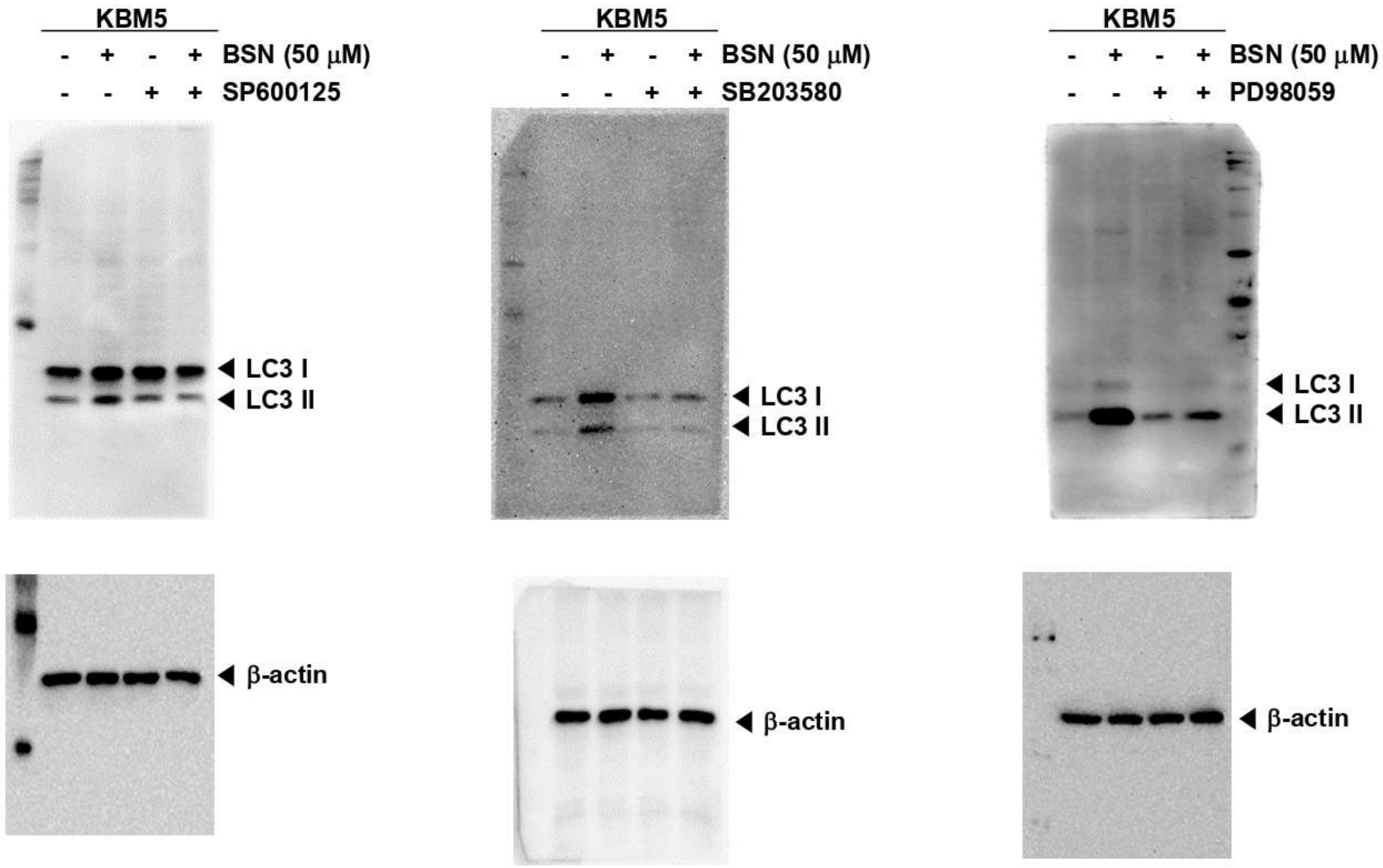
KBM5
- + - + BSN (50 μM)
- - + + SP600125
LC3 I
LC3 II
β-actin
KBM5
- + - + BSN (50 μM)
- - + + SB203580
LC3 I
LC3 II
β-actin
KBM5
- + - + BSN (50 μM)
- - + + PD98059
LC3 I
LC3 II
β-actin

Fig. S27

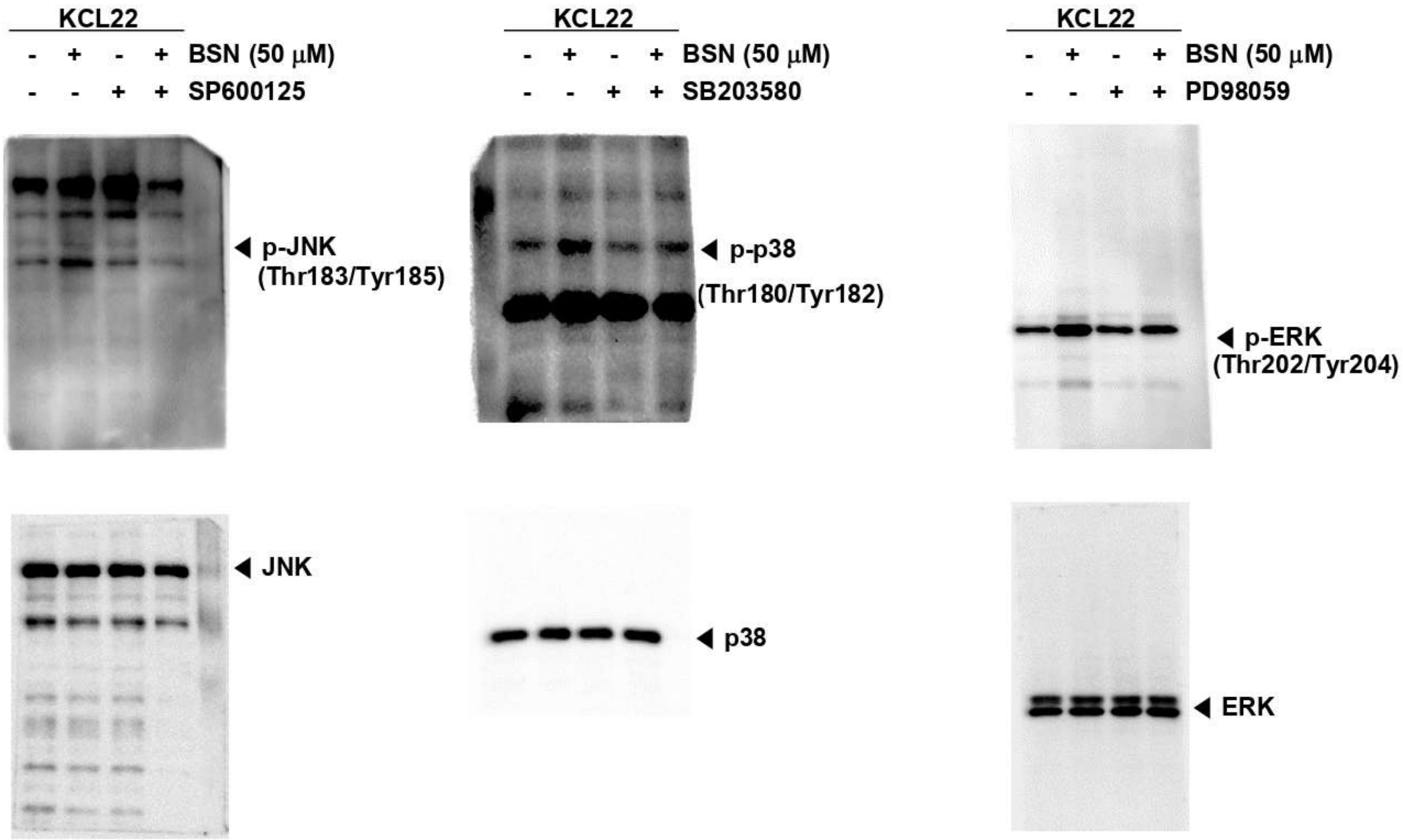
KCL22
- + - + BSN (50 μM)
- - + + SP600125
p-JNK
(Thr183/Tyr185)
JNK
KCL22
- + - + BSN (50 μM)
- - + + SB203580
p-p38
(Thr180/Tyr182)
p38
KCL22
- + - + BSN (50 μM)
- - + + PD98059
p-ERK
(Thr202/Tyr204)
ERK

Fig.S28

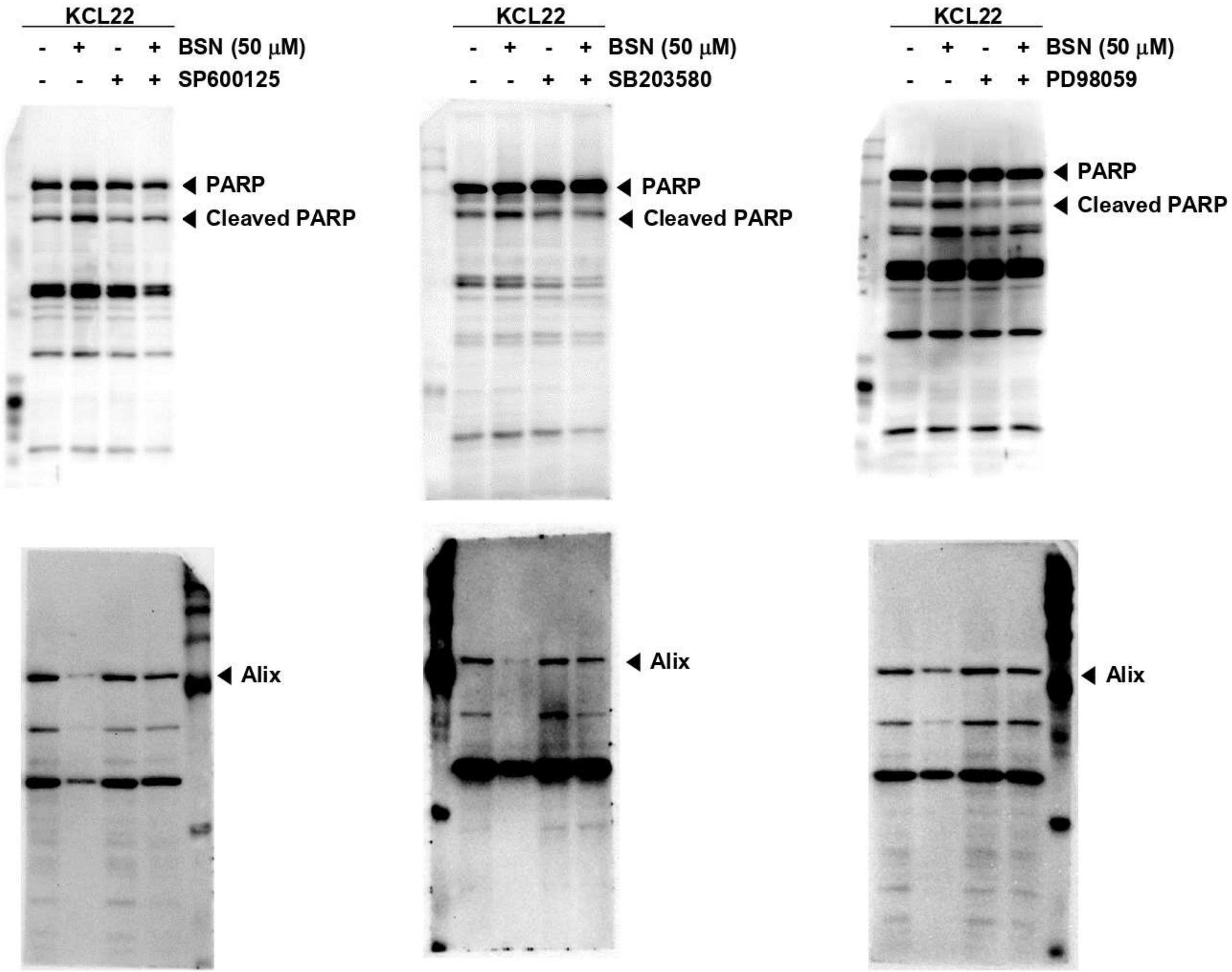
KCL22
- + - + BSN (50 μM)
- - + + SP600125
PARP
Cleaved PARP
Alix
KCL22
- + - + BSN (50 μM)
- - + + SB203580
PARP
Cleaved PARP
Alix
KCL22
- + - + BSN (50 μM)
- - + + PD98059
PARP
Cleaved PARP
Alix

Fig. S29

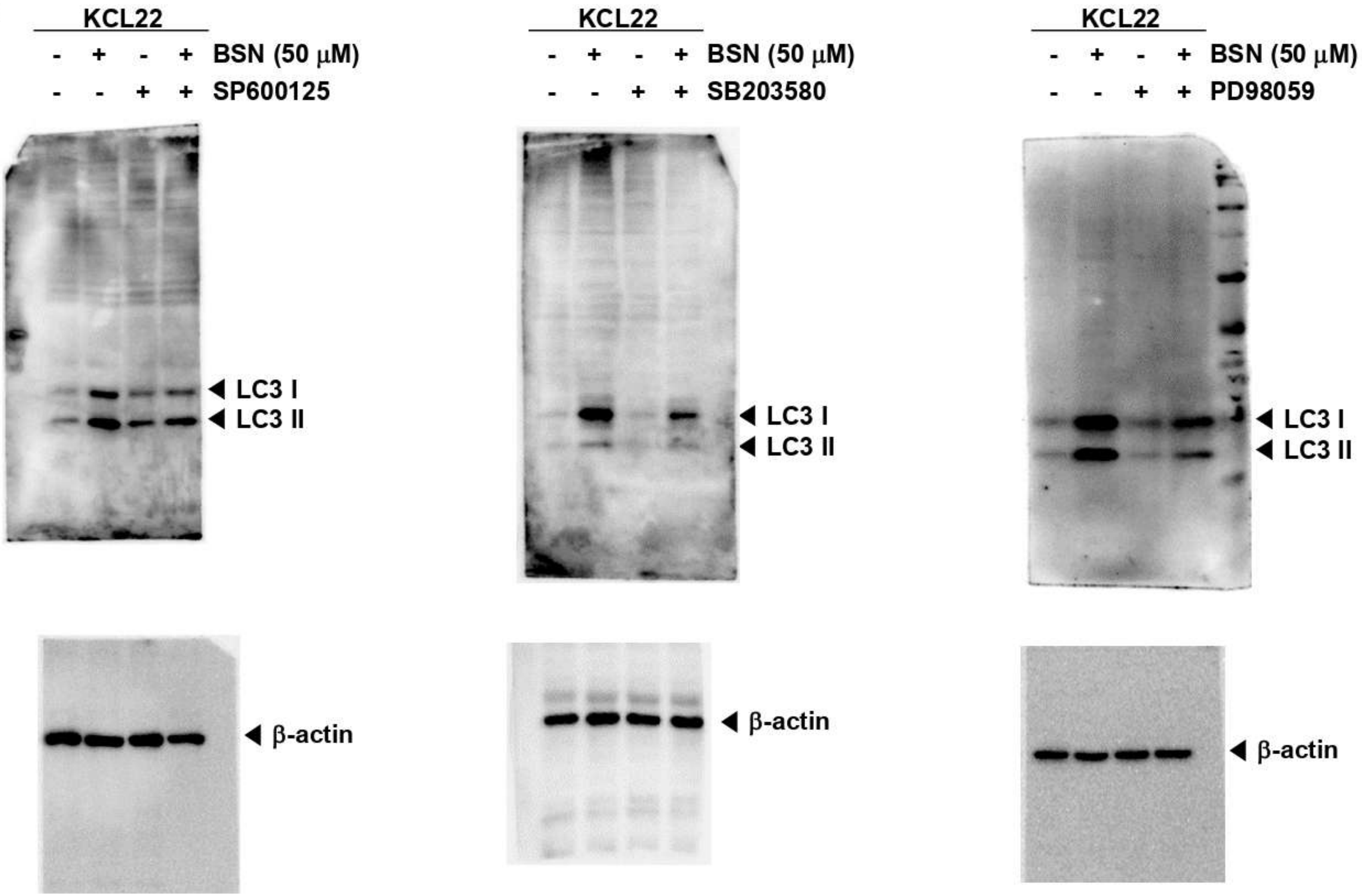
KCL22
- + - + BSN (50 μM)
- - + + SP600125
LC3 I
LC3 II
β-actin
KCL22
- + - + BSN (50 μM)
- - + + SB203580
LC3 I
LC3 II
β-actin
KCL22
- + - + BSN (50 μM)
- - + + PD98059
LC3 I
LC3 II
β-actin

Fig.S30

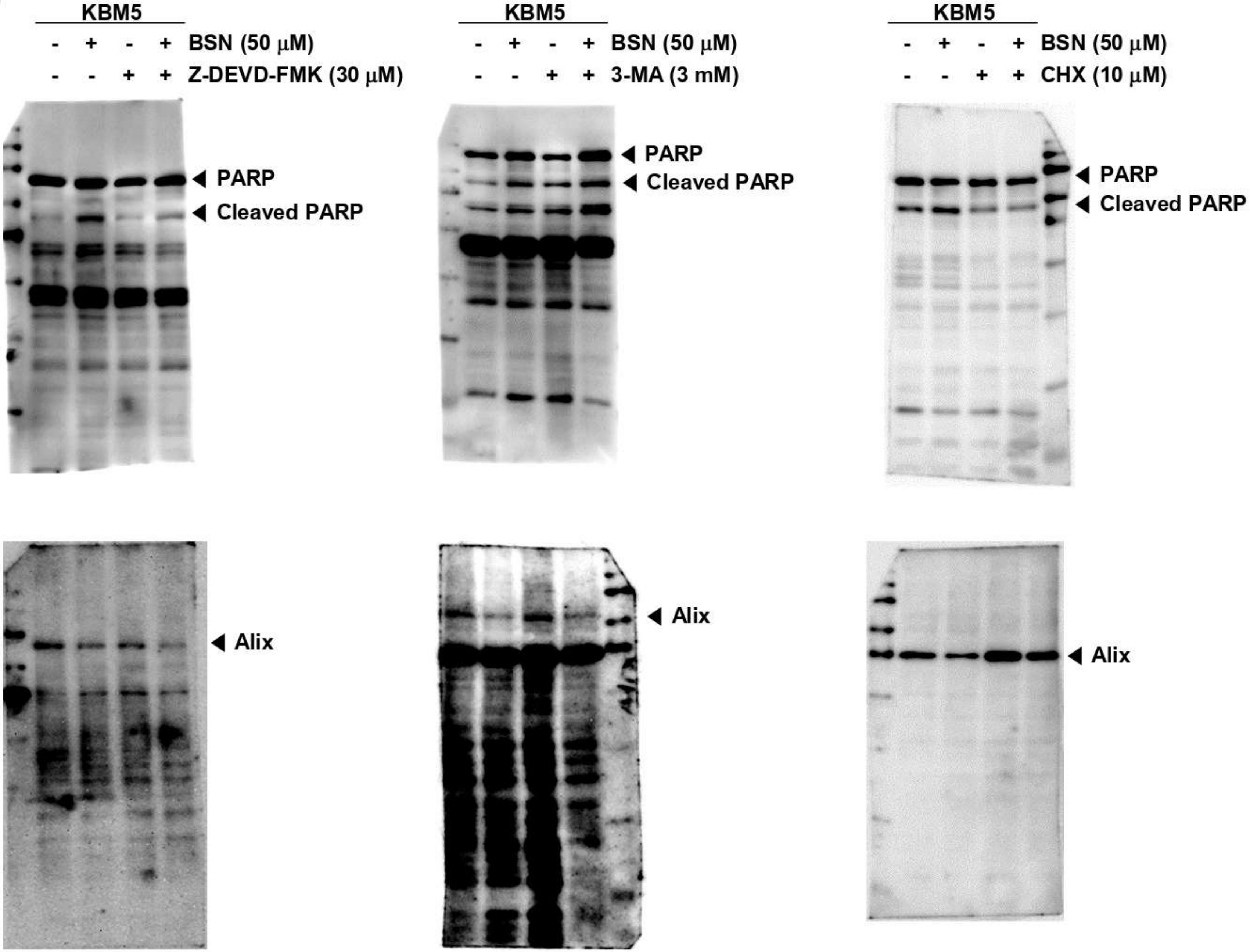
KBM5
- + - + BSN (50 μM)
- - + + Z-DEVD-FMK (30 μM)
PARP
Cleaved PARP
Alix
KBM5
- + - + BSN (50 μM)
- - + + 3-MA (3 mM)
PARP
Cleaved PARP
Alix
KBM5
- + - + BSN (50 μM)
- - + + CHX (10 μM)
PARP
Cleaved PARP
Alix

Fig. S31

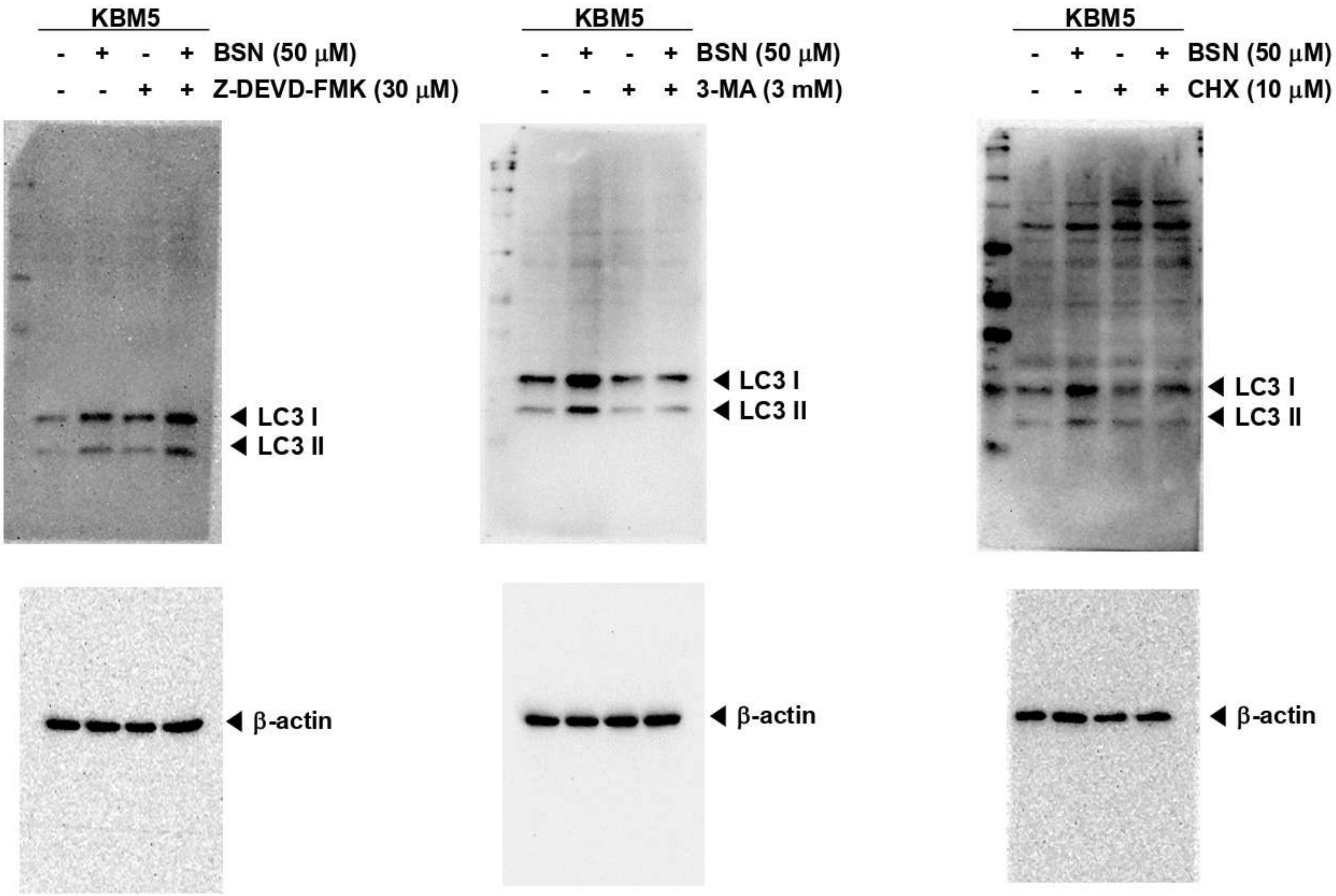

KBM5
- + - + BSN (50 μM)
- - + + Z-DEVD-FMK (30 μM)
LC3 I
LC3 II
β-actin
KBM5
- + - + BSN (50 μM)
- - + + 3-MA (3 mM)
LC3 I
LC3 II
β-actin
KBM5
- + - + BSN (50 μM)
- - + + CHX (10 μM)
LC3 I
LC3 II
β-actin
